# Preoperative estimated glomerular filtration rate to predict cardiac events in major noncardiac surgery: a secondary analysis of two large international studies

**DOI:** 10.1016/j.bja.2024.10.039

**Published:** 2025-01-02

**Authors:** Pavel S. Roshanov, Michael W. Walsh, Amit X. Garg, Meaghan Cuerden, Ngan N. Lam, Ainslie M. Hildebrand, Vincent W. Lee, Marko Mrkobrada, Kate Leslie, Matthew T.V. Chan, Flavia K. Borges, Chew Yin Wang, Denis Xavier, Daniel I. Sessler, Wojciech Szczeklik, Christian S. Meyhoff, Sadeesh K. Srinathan, Alben Sigamani, Juan Carlos Villar, Clara K. Chow, Carísi A. Polanczyk, Ameen Patel, Tyrone G. Harrison, Vikram Fielding-Singh, Juan P. Cata, Joel Parlow, Miriam de Nadal, P.J. Devereaux

**Affiliations:** 1 Department of Medicine, Western University, London, ON, Canada; 2 Population Health Research Institute, Hamilton, ON, Canada; 3 Outcomes Research Consortium, Houston, TX, USA; 4 Department of Health Research Methods, Evidence, and Impact, McMaster University, Hamilton, ON, Canada; 5 Department of Medicine, McMaster University, Hamilton, ON, Canada; 6 London Health Sciences Centre, London, ON, Canada; 7 Division of Transplantation and Nephrology, Cumming School of Medicine, University of Calgary, Calgary, AB, Canada; 8 Division of Nephrology, Department of Medicine, University of Alberta, Edmonton, AB, Canada; 9 Westmead Applied Research Centre, Faculty of Medicine and Health, University of Sydney, NSW, Australia; 10 Department of Critical Care, Melbourne Medical School, University of Melbourne, Melbourne, VIC, Australia; 11 Department of Anaesthesia and Intensive Care, The Chinese University of Hong Kong, Hong Kong, China; 12 Department of Anesthesiology, University of Malaya, Kuala Lumpur, Wilayah Persekutuan, Malaysia; 13 St John's Medical College, Bangalore, Karnataka, India; 14 Division of Clinical Research and Training, St. John's Research Institute, St. John's National Academy of Health Sciences, Bangalore, Karnataka, India; 15 Outcomes Research Consortium, Department of Anesthesiology, Critical Care and Pain Medicine, University of Texas, Houston, TX, USA; 16 Center for Intensive Care and Perioperative Medicine, Jagiellonian University Medical College, Krakow, Małopolska, Poland; 17 Department of Anaesthesia and Intensive Care, Copenhagen University Hospital - Bispebjerg and Frederiksberg, Copenhagen, Denmark; 18 Department of Surgery, University of Manitoba, Winnipeg, MB, Canada; 19 Numen Health, Bengaluru, Karnataka, India; 20 Carmel Research, Bengaluru, Karnataka, India; 21 Centro de Investigaciones, Fundación Cardioinfantil - Instituto de Cardiología, Bogotá, Colombia; 22 Facultad de Ciencias de la Salud, Universidad Autónoma de Bucaramanga, Bucaramanga, Santander, Colombia; 23 Westmead Applied Research Centre, University of Sydney, Sydney, NSW, Australia; 24 Department of Cardiology, Westmead Hospital, Sydney, NSW, Australia; 25 Graduate Program in Epidemiology and Cardiovascular Science, Federal University of Rio Grande do Sul, Porto Alegre, Rio Grande do Sul, Brazil; 26 Institute for Health Technology Assessment, Porto Alegre, Rio Grande do Sul, Brazil; 27 Department of Medicine, University of Calgary, Calgary, AB, Canada; 28 Department of Community Health Sciences, University of Calgary, Calgary, AB, Canada; 29 O'Brien Institute for Public Health, Cumming School of Medicine, University of Calgary, Calgary, AB, Canada; 30 Libin Cardiovascular Institute, Cumming School of Medicine, University of Calgary, Calgary, AB, Canada; 31 Department of Anesthesiology, Perioperative and Pain Medicine, Stanford University School of Medicine, Stanford, CA, USA; 32 Department of Anesthesiology and Perioperative Medicine, University of Texas - MD Anderson Cancer Center, Houston, TX, USA; 33 Department of Anesthesiology and Perioperative Medicine, Queen's University and Kingston Health Sciences Centre, Kingston, ON, Canada; 34 Hospital Universitari Vall d'Hebron, Universitat Autònoma de Barcelona, Barcelona, Spain

**Keywords:** chronic kidney disease, mortality, perioperative medicine, renal medicine, risk prediction, surgery, vascular events

## Abstract

**Background:**

Optimised use of kidney function information might improve cardiac risk prediction in noncardiac surgery.

**Methods:**

In 35,815 patients from the VISION cohort study and 9219 patients from the POISE-2 trial who were ≥45 yr old and underwent nonurgent inpatient noncardiac surgery, we examined (by age and sex) the association between continuous nonlinear preoperative estimated glomerular filtration rate (eGFR) and the composite of myocardial injury after noncardiac surgery, nonfatal cardiac arrest, or death owing to a cardiac cause within 30 days after surgery. We estimated contributions of predictive information, C-statistic, and net benefit from eGFR and other common patient and surgical characteristics to large multivariable models.

**Results:**

The primary composite occurred in 4725 (13.2%) patients in VISION and 1903 (20.6%) in POISE-2; in both studies cardiac events had a strong, graded association with lower preoperative eGFR that was attenuated by older age (*P*_interaction_<0.001 for VISION; *P*_interaction_=0.008 for POISE-2). For eGFR of 30 compared with 90 ml min^−1^ 1.73 m^−2^, relative risk was 1.49 (95% confidence interval 1.26–1.78) at age 80 yr but 4.50 (2.84–7.13) at age 50 yr in female patients in VISION. This differed modestly (but not meaningfully) in men in VISION (*P*_interaction_=0.02) but not in POISE-2 (*P*_interaction_=0.79). eGFR contributed the most predictive information and mean net benefit of all predictors in both studies, most C-statistic in VISION, and third most C-statistic in POISE-2.

**Conclusions:**

Continuous preoperative eGFR is among the best cardiac risk predictors in noncardiac surgery of the large set examined. Along with its interaction with age, preoperative eGFR would improve risk calculators.

**Clinical trial registration:**

ClinicalTrials.gov NCT00512109 (VISION) and NCT01082874 (POISE-2).

**Editor's key points**

• Chronic kidney disease is a risk factor for cardiovascular events and mortality, and popular preoperative cardiac risk calculators assess kidney function using a threshold for serum creatinine concentration.
• The authors performed a secondary analysis of two large prospective studies (VISION and POISE-2) to evaluate the relationship between preoperative estimated glomerular filtration rate (eGFR) and perioperative cardiac events.
• Of the large set evaluated, eGFR was one of the strongest preoperative predictors of cardiac events in noncardiac surgery.
• Future preoperative clinical risk calculators should integrate eGFR as a continuous variable along with its interaction with age in preoperative clinical risk calculators.

Chronic kidney disease is a risk factor for cardiovascular events and mortality in nonsurgical and perioperative settings.^1^, ^2^, ^3^, ^4^ Although its relationship with cardiovascular events outside of the perioperative setting is graded over a continuum of severity,^1^ popular risk calculators assess preoperative kidney function using a serum creatinine concentration above or below a threshold in perioperative cardiac risk estimation.^3^^,^^4^ Furthermore, age and sex influence the relationship between creatinine and kidney function, but it is not known if they modify the relationship between preoperative kidney function and perioperative cardiac events.^5^, ^6^, ^7^, ^8^, ^9^, ^10^, ^11^, ^12^

Estimated glomerular filtration rate (eGFR) is a measure of kidney function that is widely reported and easily calculated. The 2021 Chronic Kidney Disease Epidemiology Collaboration (CKD-EPI) creatinine equation calculates eGFR from age, sex, and serum creatinine.^13^^,^^14^ We performed secondary analyses of data from two large prospective studies (the Vascular events In noncardiac Surgery patIents cOhort evaluatioN [VISION] cohort study, and the PeriOperative Ischemic Evaluation-2 [POISE-2] randomised controlled trial [RCT]) to evaluate the relationship of preoperative eGFR and perioperative cardiac events overall and by age and sex using the 2021 race-free creatinine-based CKD-EPI equation. Our secondary objective was to compare the predictive contribution made by preoperative eGFR with that of other commonly available preoperative predictors including age, sex, preoperative haemoglobin, comorbidities, and types of surgery.

## Methods

### Study procedures

The VISION and POISE-2 studies have been described.^15^, ^16^, ^17^, ^18^

VISION (ClinicalTrials.gov: NCT00512109) was a prospective cohort study that enrolled 40,004 participants ≥45 yr old undergoing inpatient noncardiac surgery from August 2007 to October 2013 at 28 hospitals across 14 countries. Research personnel screened daily and previous-day surgical lists and patient lists in preoperative assessment clinics, in preoperative holding areas, on surgical wards, and in ICUs. Eligible consenting patients answered a series of questions about their past medical, surgical, and social history. VISION measured cardiac troponin T concentrations 6–12 h after surgery and on postoperative days 1, 2, and 3 using the Roche fourth-generation Elecsys non–high-sensitivity troponin T assay from August 2007 to January 2011, and the fifth-generation Elecsys high-sensitivity troponin T assay thereafter. VISION also obtained outcomes from medical records and a follow-up telephone interview conducted with the patient or their caregiver 30 days after surgery.

POISE-2 (ClinicalTrials.gov NCT01082874) was an RCT of 10,010 patients ≥45 yr old who had inpatient noncardiac surgery at 135 centres in 23 countries from July 2010 through December 2013. Patients were randomised in a 2×2 factorial design to perioperative aspirin or placebo and perioperative clonidine or placebo. Monitoring for myocardial injury was protocolised with measurement of cardiac injury markers 6–12 h after surgery, and on postoperative days 1, 2, and 3. Depending on local practice, these could include high- or non–high-sensitivity cardiac troponin T or I or the myocardial band of creatine kinase (CK-MB) from any test manufacturer, although CK-MB was used in only 5% and only because troponin was not available. Patients underwent electrocardiography when an elevation in cardiac markers was detected. Research personnel at participating centres followed patients up to 1 yr after randomisation, with outcomes up to 30 days after surgery reported here. The ethics review boards associated with each participating centre approved the study protocols; all participants provided informed consent.

**Fig 1:**
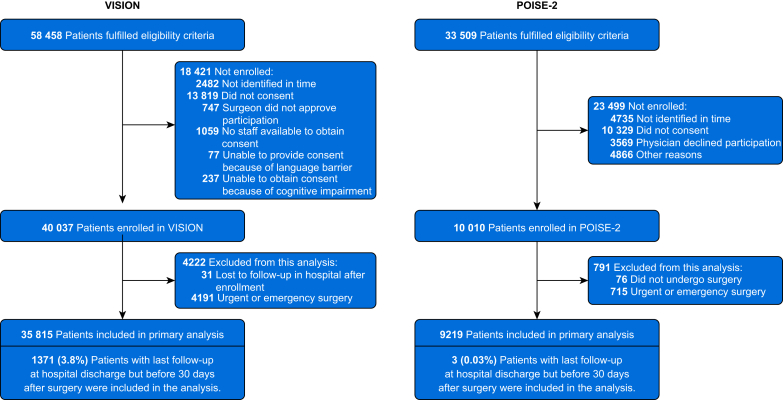
Participant flow diagram. POISE-2, PeriOperative Ischemic Evaluation 2; VISION, Vascular events In noncardiac Surgery patIents cOhort evaluatioN.

We restricted the analysis to patients undergoing nonurgent surgery to focus on the relationship between stable preoperative kidney function and perioperative cardiac outcomes to avoid including evolving preoperative acute kidney injury common in the setting of urgent or emergency surgery (Fig. 1).

### Study exposures

Supplementary material 1 defines all variables. We calculated preoperative eGFR using the 2021 CKD-EPI creatinine equation based on the most recent preoperative serum creatinine.^13^ We set a value of 0 ml min^−1^ 1.73 m^−2^ in patients receiving dialysis before surgery.

### Study outcomes

Our primary outcome was a composite of myocardial injury after noncardiac surgery (MINS) that included myocardial infarction and isolated ischaemic myocardial injury, nonfatal cardiac arrest, or death owing to a cardiac cause occurring up to 30 days after noncardiac surgery. The diagnosis of MINS that was an isolated ischaemic cardiac marker elevation was based on an elevated cardiac troponin concentration (or CK-MB concentration in some patients in POISE-2) within 30 days after noncardiac surgery in the absence of a nonischaemic aetiology (e.g. rapid atrial fibrillation, sepsis, pulmonary embolism), and without the need for an ischaemic feature (e.g. ischaemic symptom, ischaemic electrocardiography finding).^16^^,^^19^ Troponin elevations without other features of myocardial ischaemia to meet the definition of myocardial infarction were centrally adjudicated by perioperative physicians for potential nonischaemic aetiology in VISION. In POISE-2, we identified from the study database atrial fibrillation, flutter, pulmonary embolism, or sepsis that occurred within 48 h of the time that the troponin elevation first met the specified MINS criteria and automatically refuted those MINS events. Other outcomes were adjudicated centrally.

### Variables included for adjustment

We adjusted the analyses for other patient and surgical characteristics. In the analyses of VISION, these included age, sex, preoperative haemoglobin, the need for assistance with activities of daily living, history of congestive heart failure, coronary artery disease, peripheral vascular disease, stroke or transient ischaemic attack, hypertension, diabetes mellitus (as a categorical variable including ‘no diabetes’, ‘diabetes not treated with preoperative insulin’, and ‘diabetes treated with preoperative insulin’), history of atrial fibrillation, active cancer, history of chronic obstructive pulmonary disease, 25 types of surgery, and planned use of an open (*vs* endoscopic) surgical approach. The analyses of POISE-2 used a similar but slightly smaller set of variables.

### Statistical analysis

We performed the analyses using R version 4.3 (R Foundation for Statistical Computing, Vienna, Austria).

### Primary analysis

We used modified Poisson regression models to estimate the independent association (as an adjusted relative risk, aRR) between the primary composite outcome and eGFR, with 95% confidence intervals (CIs) using a cluster-robust variance estimator where study centres defined the clusters.^20^^,^^21^ We modelled eGFR as a continuous variable using restricted cubic splines.^22^ We included multiplicative interaction terms for eGFR-by-sex and eGFR-by-age (where age was modelled with restricted cubic splines), and performed two-sided statistical tests for interaction to determine if age or sex modified the relationship between eGFR and cardiac events. We placed knots at 110, 90, 60, and 30 ml min^−1^ 1.73 m^−2^ for eGFR and at 50, 65, and 80 yr of age. Before conducting the analyses, we chose the number of knots and their locations with consideration of the distribution of patients and outcome events, where we might see changes in the shape of the relationship, and of the demands placed on the data by interaction terms that multiply the number of parameters estimated for these splines.

To aid with interpretation, we additionally presented results of the primary analysis in model-predicted absolute risks from logistic regression models fit with the same variables and the same cluster-robust variance estimator. *P*<0.05 denoted statistical significance for all analyses; we kept all variables in the models regardless of statistical significance.

### Prespecified secondary analyses

We assessed the predictive importance of preoperative eGFR relative to other predictors. Firstly, we estimated the percentage of total predictive information contributed by each predictor variable to a logistic regression model.^23^^,^^24^ This value was calculated as the likelihood ratio χ^2^ statistic attributable to a variable after subtracting the degrees of freedom spent in modelling that variable, divided by the total likelihood ratio χ^2^ statistic for the model, multiplied by 100. A higher value indicates greater contribution to the total model predictive information. Secondly, we estimated the absolute gain in C-statistic attributable to each variable. Thirdly, we conducted decision curve analysis to estimate net benefit^25^ in terms of the net number of true positives gained per 1000 patients at minimal clinically important risk thresholds 10%, 20%, 30%, and 40%. We calculated the mean difference in net benefit curve across these thresholds. We conducted these analyses across 1000 cluster-based bootstrap samples; the median value provided the point estimate, and the 2.5th and 97.5th percentile values formed the 95% CI. We also internally validated with bootstrapping the logistic regression models in each study to ensure that they served as a robust basis for these analyses.

### Sensitivity analyses

First, we repeated the primary analysis only in patients without missing data. Second, we repeated the primary and secondary analyses but modified the composite outcome to include only myocardial infarction, nonfatal cardiac arrest, or death owing to a cardiac cause (i.e. excluding MINS that was isolated ischaemic myocardial injury). Third, we repeated the primary and secondary analyses but only included patients with eGFR ≥30 ml min^−1^ 1.73 m^−2^ because previous analyses have shown a diminished relationship between fourth-generation troponin T and perioperative mortality in patients with eGFR <30 ml min^−1^ 1.73 m^−2^.^26^ Fourth, we repeated the primary analysis using logistic regression to estimate odds ratios instead of relative risks. At a reviewer's request, we repeated the primary and secondary analyses in patients who were not receiving dialysis before surgery. We present several figures to inform the extent to which adding eGFR affects predicted probabilities across age, sex, and eGFR value.

### Approach to missing data

We imputed missing data in both cohorts separately using multiple imputation by chained equations (20 imputed datasets each) with random forests for the primary analysis.^27^ Imputation models included the primary outcome and all covariates.^28^ We performed secondary and sensitivity analyses in patients without missing data except for estimation of odds ratios which we performed after imputation.

### Sample size

We estimated sample size requirements for the primary analysis to avoid overestimation of effects using the R software package pmsampsize.^29^ In VISION and POISE-2, respectively, we considered 56 and 38 model parameters, known outcome incidences of 13.2% and 20.6%, a conservative estimate for expected model performance (Cox-Snell R^2^ of 10%), and a stringent requirement for minimal overestimation of regression coefficients (shrinkage 0.95). In VISION, we required 12 728 patients and 1681 events for the primary analysis and performed it in 35 815 patients with 4725 events. In POISE-2, we required 8637 patients and 1780 events for the primary analysis and performed it in 9219 patients with 1903 events.

## Results

**Fig 2:**
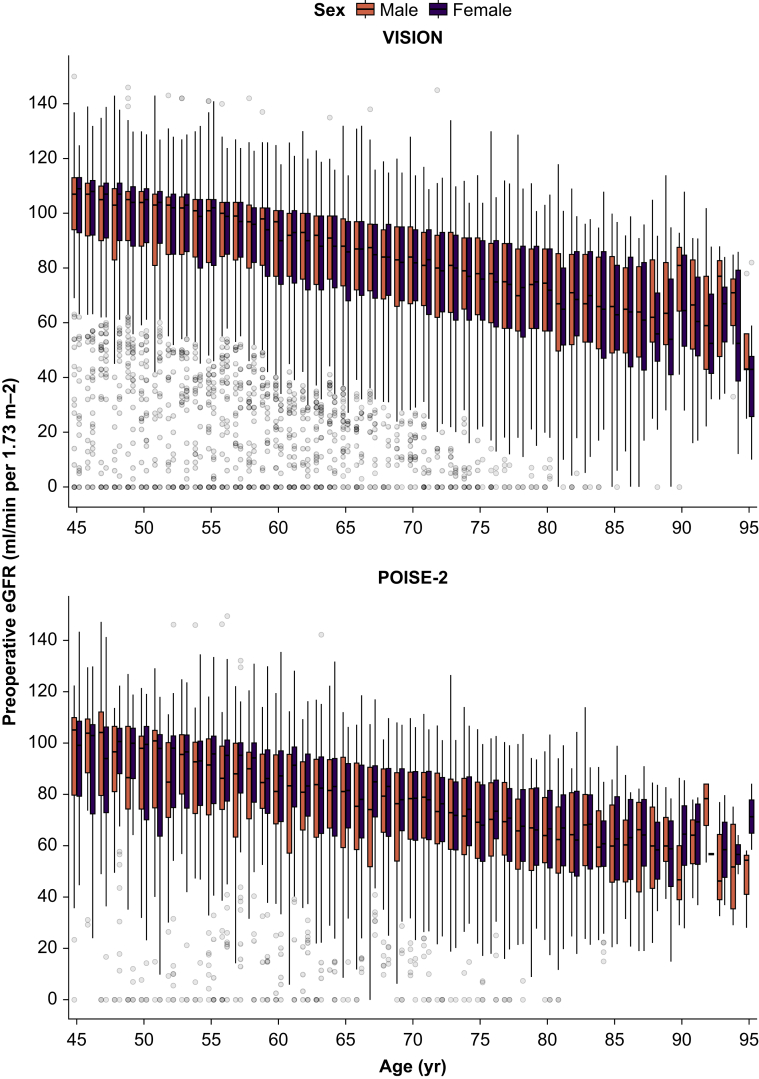
**Relationship between age, sex, and preoperative kidney function.** eGFR, estimated glomerular filtration rate; POISE-2, PeriOperative Ischemic Evaluation 2; VISION, Vascular events In noncardiac Surgery patIents cOhort evaluatioN.

Table 1 provides an abridged summary of the VISION and POISE-2 cohorts. Detailed cohort summaries of variables included in the models can be found in Tables S1 and S2 in Supplementary material 1. In VISION and POISE-2, respectively, patients had a median age of 63 yr (interquartile range [IQR] 54–72) and 70 yr (61–76), females were 49.6% and 46.8%, and the median eGFR was 89 ml min^−1^ 1.73 m^−2^ (IQR 71–101) and 82 ml min^−1^ 1.73 m^−2^ (IQR 64–94); 17.6% in VISION and 21.7% in POISE-2 had eGFR <60 ml min^−1^ 1.73 m^−2^, and 1.4% in VISION and 1.2% in POISE-2 were receiving dialysis before surgery. The eGFR decreased with age similarly for males and females in both studies (Fig. 2). Orthopaedic and general surgeries accounted for more than half of the surgeries in both studies. Data regarding at least one variable (mostly preoperative serum creatinine and haemoglobin) were missing in 2643 patients (7.4%) before imputation in VISION and in 326 (3.5%) in POISE-2.

**Table 1:** Participant characteristics and outcomes. Data are presented before imputation. Continuous variables are presented as median (25th percentile–75th percentile); categorical variables are presented as number (% of study total). ∗Missing data in VISION: 2643 (7.4%) in total; estimated glomerular filtration rate (owing to missing serum creatinine), 2281 (6.4%); preoperative haemoglobin, 1235 (3.4%); open surgery, 22 (0.1%). Missing data on variables not shown here: hypertension, 31 (0.1%); history of congestive heart failure, 50 (0.1%); history of atrial fibrillation, 87 (0.2%); diabetes mellitus, 26 (0.1%); requires help with activities of daily living, 60 (0.2%). ^†^Missing data in POISE-2: 326 (3.5%) in total; estimated glomerular filtration rate (owing to missing serum creatinine), 136 (1.5%); preoperative haemoglobin, 235 (2.5%); in atrial fibrillation before surgery, not shown here, 1 (<0.1%). MINS, myocardial injury after noncardiac surgery; POISE-2, PeriOperative Ischemic Evaluation 2; VISION, Vascular events In noncardiac Surgery patIents cOhort evaluatioN.

	VISION∗	POISE-2^†^
	*N*=35,815	*N*=9219
**Age, yr**	63 (54–72)	70 (61–76)
**Female**	17,767 (49.6)	4310 (46.8)
**Male**	18,048 (50.4)	4909 (53.2)
**Preoperative haemoglobin (g L^−1^)**	132 (120–144)	133 (121–144)
**Preoperative estimated glomerular filtration rate (ml min^−1^ 1.73 m^−2^)**	89 (71–101)	82 (64–94)
**Preoperative dialysis**	485 (1.4)	108 (1.2)
**History of hypertension**	18,217 (50.9)	8098 (87.8)
**History of coronary artery disease**	4730 (13.2)	2167 (23.5)
**History of peripheral arterial disease**	2910 (8.1)	807 (8.8)
**History of stroke or transient ischaemic attack**	2225 (6.2)	802 (8.7)
**History of congestive heart failure**	1243 (3.5)	305 (3.3)
**Major orthopaedic surgery**	6012 (16.8)	3283 (35.6)
**Major general surgery**	6836 (19.1)	1692 (18.4)
**Major thoracic surgery**	1112 (3.1)	394 (4.3)
**Major urogenital surgery**	4655 (13.0)	1190 (12.9)
**Major vascular surgery**	2,508 (7.0)	477 (5.2)
**Major spine surgery**	1,358 (3.8)	193 (2.1)
**Open surgery**	27,723 (77.5)	7126 (77.3)
**Outcomes within 30 days after surgery**		
**MINS, nonfatal cardiac arrest, or death owing to cardiac cause**	4725 (13.2)	1903 (20.6)
**Myocardial infarction, nonfatal cardiac arrest, or death owing to cardiac cause**	1174 (3.3)	613 (6.6)
**MINS**	4670 (13.0)	1881 (20.4)
**Isolated ischaemic myocardial injury**	3582 (10.0)	1307 (14.2)
**Myocardial infarction**	1088 (3.0)	574 (6.2)
**Nonfatal cardiac arrest**	44 (0.1)	18 (0.2)
**Death owing to cardiac cause**	102 (0.3)	56 (0.6)

### Relationship between preoperative eGFR and perioperative cardiac events

**Fig 3:**
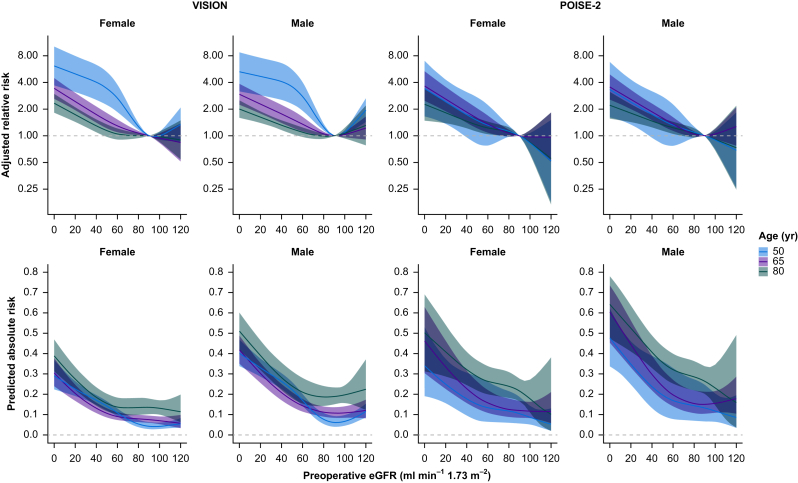
Age- and sex-specific relationship between preoperative kidney function and perioperative cardiac events. Results from regression models for the composite of MINS, nonfatal cardiac arrest, or death owing to a cardiac cause occurring within 30 days after nonurgent noncardiac surgery in 35,815 patients in VISION and 9219 patients in POISE-2, pooled across 20 imputed datasets. The top panels show the relative risk across eGFR in female and male patients aged 50, 65 and 80 yr in modified Poisson regression. The reference eGFR was 90 ml min^−1^ 1.73 m^−2^. eGFR was associated with the composite outcome (*P*<0.001) in a nonlinear fashion (*P*_nonlinearity_<0.001) and differed by age (*P*_interaction_<0.001) and modestly by sex (*P*_interaction_=0.02). In POISE-2, the association was not significantly nonlinear (*P*_nonlinearity_=0.33) and differed by age (*P*_interaction_=0.008) but not sex (*P*_interaction_=0.79). The bottom panels show absolute risk predictions for hypothetical patients undergoing hip surgery who had a preoperative haemoglobin of 13.2 g L^−1^ and no other comorbidities, predicted from logistic regression models. eGFR, estimated glomerular filtration rate; MINS, myocardial injury after noncardiac surgery; POISE-2, PeriOperative Ischemic Evaluation 2; VISION, Vascular events In noncardiac Surgery patIents cOhort evaluatioN.

The primary composite of 30-day MINS, nonfatal cardiac arrest, or death owing to a cardiac cause occurred in 4725 patients (13.2%) in VISION and 1903 (20.6%) in POISE-2. Figure 3 shows a graded relationship where lower eGFR associates with a higher risk of the primary outcome in both studies (*P*<0.001). In VISION, the relationship was not linear (*P*_nonlinearity_<0.001) and differed by age (*P*_interaction_<0.001) such that the aRR of cardiac events began to increase at a lower eGFR in older patients (e.g. at ∼45 ml min^−1^ 1.73 m^−2^ for patients aged 80 yr) compared with younger patients (e.g. at ∼85 ml min^−1^ 1.73 m^−2^ for patients aged 50 yr). Compared with an eGFR of 90 ml min^−1^ 1.73 m^−2^ in VISION, the aRR between eGFR and cardiac events was 1.05 (95% CI 0.91–1.21) at an eGFR of 60 ml min^−1^ 1.73 m^−2^ and 1.49 (1.26–1.78) at an eGFR of 30 ml min^−1^ 1.73 m^−2^ in an 80-yr-old female patient. Conversely, the aRR was 2.77 (1.92–4.02) at an eGFR of 60 ml min^−1^ 1.73 m^−2^ and 4.50 (2.84–7.13) at an eGFR of 30 ml min^−1^ 1.73 m^−2^ in a 50-yr-old female patient. Although modestly different between male and female patients quantitatively (*P*_interaction_=0.02), this relationship was qualitatively similar. In POISE-2, the relationship between eGFR and the primary composite outcome was more linear (*P*_nonlinearity_=0.33) and differed similarly by age (*P*_interaction_=0.008), but not by sex (*P*_interaction_=0.79).

Figure 3 also presents predicted absolute risks. Based on the VISION model, a hypothetical 50-yr-old female patient who underwent hip surgery with a preoperative haemoglobin of 13.2 g dl^−1^ and none of the comorbidities captured in that model would have 4.2% (95% CI 2.8–6.3%) predicted risk of the primary composite outcome at an eGFR of 90 ml min^−1^ 1.73 m^−2^. However, that risk would be 19.7% (95% CI 17.2–22.5%) at an eGFR of 30 ml min^−1^ 1.73 m^−2^. This approaches the 22.6% (95% CI 18.0–27.9%) risk of an 80-yr-old female patient with an eGFR of 30 ml min^−1^ 1.73 m^−2^ undergoing the same surgery with an otherwise identical profile, and surpasses the 13.6% (95% CI 10.2–17.9%) risk in a similar 80-yr-old female patient with an eGFR of 90 ml min^−1^ 1.73 m^−2^. Predicted risks were ∼1.4-times higher in male patients. Similarly, in POISE-2, a 50-yr-old female patient with an eGFR of 30 ml min^−1^ 1.73 m^−2^ had a predicted absolute risk of 21.0% (95% CI 12.6–33%), approaching the 21.6% (95% CI 16.6–27.7%) predicted for an 80-yr-old patient with an eGFR of 90 ml min^−1^ 1.73 m^−2^ and an otherwise identical profile.

### Relative importance of eGFR in risk prediction

**Fig 4:**
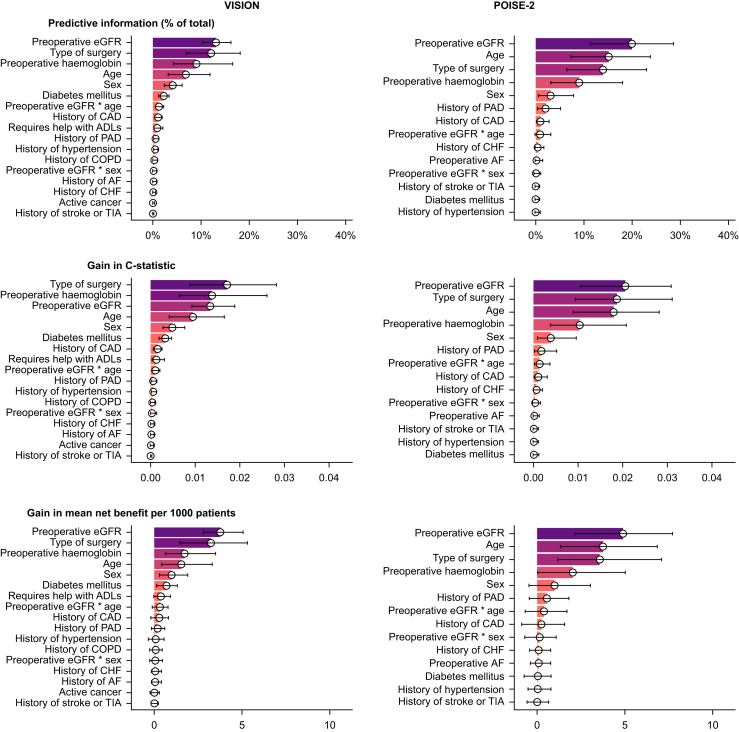
Predictor importance for the composite of MINS, nonfatal cardiac arrest, or death owing to a cardiac cause after nonurgent noncardiac surgery. Percentage of total predictive information, gain in C-statistic, and gain in mean net benefit contributed by individual predictors from logistic regression models predicting a composite of MINS, nonfatal cardiac arrest, or death owing to a cardiac cause occurring within 30 days after nonurgent noncardiac surgery in patients without missing data in VISION (*n*=33 171) and POISE-2 (*n*=8893). The contributions from all surgical variables are summed in a single ‘Type of Surgery’ item for clarity. Point estimates (medians) and 95% confidence intervals are derived from 1000 cluster-based bootstrap samples. ADLs, activities of daily living; AF, atrial fibrillation; CAD, coronary artery disease; CHF, congestive heart failure; COPD, chronic obstructive pulmonary disease; eGFR, estimated glomerular filtration rate; MINS, myocardial injury after noncardiac surgery; PAD, peripheral arterial disease; POISE-2, PeriOperative Ischemic Evaluation 2; TIA, transient ischaemic attack; VISION, Vascular events In noncardiac Surgery patIents cOhort evaluatioN.

In both studies, preoperative eGFR provided the greatest percentage of total predictive information and the largest gain in mean net benefit for the primary outcome (Fig. 4). It ranked first for gain in C-statistic in POISE-2 and third in VISION. The other important predictors were older age, lower preoperative haemoglobin concentration, and type of surgery. The interaction between eGFR and age ranked similarly in importance to history of coronary artery disease, but the interaction with sex was consistently among the least important predictors. Figures S1 and S2 in Supplementary material 1 show acceptable performance in the internal validation of the VISION and POISE-2 models used for these analyses.

### Sensitivity analyses

Figure S3 in Supplementary material 1 shows results of sensitivity analyses of the association between preoperative eGFR and cardiac events. Results remained consistent with the main analyses after excluding patients with missing data. The interaction between eGFR and age remained consistent for both studies when we modified the composite outcome to include only myocardial infarction, nonfatal cardiac arrest, or death owing to a cardiac cause, but the interaction with sex was no longer significant in either study. In analyses restricted to eGFR ≥30 ml min^−1^ 1.73 m^−2^ and restricted to patients not receiving dialysis before surgery, the results remained qualitatively similar, but the interaction with sex was no longer significant in either study. In the analyses that modelled odds ratios instead of relative risks, the results remained qualitatively similar, but the interaction with sex was no longer significant in either study, and the interaction with age was no longer significant in POISE-2. Preoperative eGFR remained among the most important predictors of cardiac events across sensitivity analyses (Figs S4, S5, and S6 in Supplementary material 1).

### Additional descriptive analyses

Figure S7 in Supplementary material 1 shows the extent to which adding eGFR affected predicted probabilities of the primary outcome. Figure S8 shows that accounting for eGFR when eGFR <50 ml min^−1^ 1.73 m^−2^ substantially increases the predicted risk while moderately reducing the predicted risk for a small proportion of participants with eGFR >50 ml min^−1^ 1.73 m^−2^. For a small proportion of male, but not female, participants in VISION, eGFR >100 ml min^−1^ 1.73 m^−2^ increased risk estimates which might explain the interaction between eGFR and sex found in VISION but not in POISE-2. Figure S9 shows that the distribution of change in predicted probability when eGFR is added to the model is largely consistent across age in both studies until ∼age 80 yr. Adding eGFR meaningfully changed the predicted probability in fewer patients older than 80 yr, consistent with the interaction between eGFR and age.

## Discussion

Preoperative eGFR was a strong predictor of perioperative cardiac events in two large international prospective studies of patients aged ≥45 yr who had nonurgent inpatient noncardiac surgery. It was consistently among the most informative predictors of perioperative cardiac events, surpassing both age and history of cardiovascular disease. The relative impact of preoperative eGFR on perioperative cardiac risk estimates was greater in younger people than in older people, but qualitatively similar for male and female patients. Moreover, we identified lower preoperative haemoglobin and advanced age, which often coexist with low eGFR, as additional strong independent predictors of perioperative cardiac events.

Our evaluation shows that eGFR modelled as a continuous nonlinear function makes a substantial contribution of predictive information to a large base model with optimally modelled continuous predictors. Although we did not directly compare the performance with that of existing models, they have modest performance,^30^ are less comprehensive, and treat eGFR in an inferior way as a binary predictor even though the relationship between eGFR and cardiac events does not have a threshold appropriate for dichotomisation. On this basis, we believe it is reasonable to expect that modelling eGFR in this way would improve on other models. Risk prediction systems that dichotomise kidney function at single thresholds waste information and are arguably less useful as a simplification in the era of mobile devices.^3^^,^^4^^,^^22^^,^^31^, ^32^, ^33^

Best statistical use of eGFR for prediction would also account for its interaction with age. Preoperative kidney function was more strongly associated with perioperative cardiac risk in younger patients compared with older patients, particularly after 80 yr of age, consistent with the concept that nephrosclerosis and loss of functional glomeruli accompany normal ageing.^34^ eGFR remains informative in older patients because, although its relative effect on predicted risk is attenuated, the absolute effect remains large given their higher baseline risk. Although it could also be considered, the interaction with sex was not consistent across studies and, when found, was modest and qualitatively unimportant.

### Limitations

Both studies collected the single most recent preoperative serum creatinine measurement, and some patients might have had acute kidney injury at the time of measurement. However, practitioners commonly rely on the most recent creatinine value, and we excluded urgent surgeries because preoperative acute kidney injury is most likely to occur in this setting. Neither study collected urinary protein which predicts cardiovascular events independently of eGFR in nonsurgical studies.^1^

We required a common reference point on the eGFR continuum (90 ml min^−1^ 1.73 m^−2^) to facilitate comparison of relative risks across ages. Our chosen reference point might have underestimated or overestimated the relative risk at lower eGFR for younger patients compared with a higher reference point, but estimates were uncertain at higher eGFR. The models do not inherently have a reference point, so the choice of reference point does not affect any of the assessments of added predictive information or the tests of interaction.

### Conclusions

Commonly available eGFR is one of the strongest predictors of cardiac events in noncardiac surgery of the large set evaluated. Its integration into clinical risk calculators as a continuous variable along with its interaction with age warrants consideration.

## Author's contributions

Conception of secondary analyses: PSR

Principal investigator of the analysed studies: PJD

Acquisition, analysis, or interpretation of data: all authors

Drafting of the manuscript: PSR

Critical revision of the manuscript for important intellectual content: all authors

Statistical analysis: PSR, MC

PSR had full access to all the data in the study and takes responsibility for the integrity of the data and the accuracy of the data analysis.

## Funding

The POISE-2 and VISION funding sources had no role in the design and conduct of the study; collection, management, analysis, and interpretation of the data; preparation, review, or approval of the manuscript; or the decision to submit the manuscript for publication. The POISE-2 study was funded by the Canadian Institutes of Health Research, the National Health and Medical Research Council of Australia, and the Spanish Ministry of Health and Social Policy. Bayer Pharma provided the aspirin used in the POISE-2 study, and Boehringer Ingelheim provided the clonidine and some financial support for the POISE-2 study. Roche Diagnostics provided the troponin T assays as well as financial support for the VISION study. Funding for the VISION study came from more than 70 grants: Canada: Canadian Institutes of Health Research (seven grants); Heart and Stroke Foundation of Ontario (two grants); Academic Health Science Centres Alternative Funding Plan Innovation Fund Ontario; Population Health Research Institute; CLARITY Research Group; McMaster University Department of Surgery Surgical Associates; Hamilton Health Science New Investigator Fund; Hamilton Health Sciences; Ontario Ministry of Resource and Innovation; Stryker Canada; McMaster University, Department of Anesthesiology (two grants); St Joseph's Healthcare, Department of Medicine (two grants); Father Sean O'Sullivan Research Centre (two grants); McMaster University Department of Medicine (two grants); Roche Diagnostics Global Office (five grants); Hamilton Health Sciences Summer Studentships (six grants); McMaster University Department of Clinical Epidemiology and Biostatistics; McMaster University, Division of Cardiology; Canadian Network and Centre for Trials Internationally; Winnipeg Health Sciences Foundation; University of Manitoba Department of Surgery (two grants); Diagnostic Services of Manitoba Research; Manitoba Medical Services Foundation; Manitoba Health Research Council; University of Manitoba Faculty of Dentistry Operational Fund; University of Manitoba Department of Anesthesia; University Medical Group, Department of Surgery, University of Manitoba, Start-up Fund. Australia: National Health and Medical Research Council Program. Brazil: Projeto Hospitais de Excelência a Serviço do SUS (PROADI-SUS) grant from the Brazilian Ministry of Health in partnership with Hcor (Cardiac Hospital Sao Paulo–SP); National Council for Scientific and Technological Development (CNPq) grant from the Brazilian Ministry of Science and Technology. China: Public Policy Research Fund (grant CUHK-4002-PPR-3), Research Grant Council, Hong Kong SAR; General Research Fund (grant 461412), Research Grant Council, Hong Kong SAR; Australian and New Zealand College of Anaesthetists (grant 13/008). Colombia: School of Nursing, Universidad Industrial de Santander; Grupo de Cardiología Preventiva, Universidad Autónoma de Bucaramanga; Fundación Cardioinfantil–Instituto de Cardiología; Alianza Diagnóstica SA. France: Université Pierre et Marie Curie, Département d'anesthésie Réanimation, Pitié-Salpêtrière, Assistance Publique–Hôpitaux de Paris. India: St John's Medical College and Research Institute; Division of Clinical Research and Training. Malaysia: University of Malaya (grant RG302-14AFR); University of Malaya, Penyelidikan Jangka Pendek. Poland: Polish Ministry of Science and Higher Education (grant NN402083939). South Africa: University of KwaZulu-Natal. Spain: Instituto de Salud Carlos III; Fundació La Marató de TV3. USA: American Heart Association; Covidien; American Society of Nephrology Student Scholar Grant. UK: National Institute for Health Research. Pavel Roshanov is recipient of a career award from the Academic Medical Organization of Southwestern Ontario and his research is supported by the William F. Clark Chair in Nephrology from Western University. Flavia Borges is recipient of an Early Career Research Award from Hamilton Health Sciences. Tyrone Harrison is supported by a Kidney Research Scientist Core Education and National Training (KRESCENT) Program New Investigator Award (co-sponsored by the Kidney Foundation of Canada and Canadian Institutes of Health Research) and is supported as a new investigator by the Roy and Vi Baay Chair for Kidney Research and the Kidney Health and Wellness Institute at the University of Calgary. Vikram Fielding-Singh has received support from the American Heart Association (23CDA1053913) and the National Institute of Diabetes and Digestive and Kidney Diseases of the National Institutes of Health (K23DK138312).

## Declarations of interest

Roche Diagnostics provided troponin T assays and financial support for the VISION study. Bayer Pharma provided the aspirin used in the POISE-2 study, and Boehringer Ingelheim provided the clonidine and financial support for the POISE-2 study. Dr Devereaux reports grants from Roche Diagnostics and Abbott Diagnostics during the conduct of the study, and grants from Roche, Octopharma, Philips Healthcare, Stryker, Covidien, and Boehringer Ingelheim outside the submitted work. Dr Meyhoff is founder of a start-up company, WARD24/7 ApS, with the aim of pursuing the regulatory and commercial activities of the WARD-project (Wireless Assessment of Respiratory and circulatory Distress, a project developing a clinical support system for continuous wireless monitoring of vital signs) and has filed a patent for ‘Wireless Assessment of Respiratory and circulatory Distress (WARD), EP 21184712.4 and EP 21205557.8’. Dr Borges reports grants from Roche Diagnostics outside the scope of this work.
