# Supplementary material for: Preoperative estimated glomerular filtration rate to predict cardiac events in major noncardiac surgery: a secondary analysis of two large international studies
Source: Br J Anaesth. 2025 Jan 2;134(2):297–307. doi: 10.1016/j.bja.2024.10.039 (PMC11775841; doi:10.1016/j.bja.2024.10.039)
Supplement: Multimedia component 1 [file mmc1.docx]

**Supplement 1**

for

Preoperative estimated glomerular filtration rate to predict cardiac events in major noncardiac surgery.

Roshanov PS *et al.*

**Table of Contents**

[Study eligibility criteria 2](#_Toc179809152)

[VISION eligibility criteria 2](#_Toc179809153)

[POISE-2 eligibility criteria 2](#_Toc179809154)

[VISION variable definitions 3](#_Toc179809155)

[Outcome definitions 3](#_Toc179809156)

[Type of surgery performed 4](#_Toc179809157)

[Patient factors 5](#_Toc179809158)

[POISE-2 variable definitions 7](#_Toc179809159)

[Outcome definitions 7](#_Toc179809160)

[Type of surgery performed 8](#_Toc179809161)

[Patient factors 9](#_Toc179809162)

[eTable 1. VISION participant characteristics. 11](#_Toc179809163)

[eTable 2. POISE-2 participant characteristics. 12](#_Toc179809164)

[eFigure 1. Internal validation of the primary VISION prediction model. 13](#_Toc179809165)

[eFigure 2. Internal validation of the primary POISE-2 prediction model. 14](#_Toc179809166)

[eFigure 3. Sensitivity analyses of age-and-sex-specific relationship between preoperative eGFR and perioperative cardiac events. 15](#_Toc179809167)

[eFigure 4. Predictor importance for composite outcome of myocardial infarction, nonfatal cardiac arrest, or death due to cardiac cause. 17](#_Toc179809168)

[eFigure 5. Predictor importance restricted to patients with eGFR ≥ 30 ml/min per 1.73 m^2^. 18](#_Toc179809169)

[eFigure 6. Predictor importance restricted to patients not receiving dialysis before surgery. 19](#_Toc179809170)

[eFigure 7. Relationship between predicted probability of cardiac events with versus without eGFR in the model, by sex. 20](#_Toc179809171)

[eFigure 8. Change in predicted probability of perioperative cardiac events with addition of eGFR to the model across the range of eGFR, by sex. 21](#_Toc179809172)

[eFigure 9. Change in predicted probability of perioperative cardiac events with addition of eGFR to the model across age, by sex. 22](#_Toc179809173)

# Study eligibility criteria

##

## VISION eligibility criteria

1. ≥45 years old
2. Having noncardiac surgery with at least overnight hospital stay
3. Not previously enrolled in VISION
4. Undergoing non-urgent, non-emergency surgery (applies to this analysis only)

## POISE-2 eligibility criteria

**Inclusion criteria (any of)**

1. History of coronary artery disease
2. History of peripheral arterial disease
3. History of stroke
4. Undergoing major vascular surgery (i.e., all vascular surgery except for: arteriovenous shunts for dialysis, vein stripping procedures, carotid endarterectomies and endovascular abdominal aortic aneurysm repair)
5. Undergoing intraperitoneal, intrathoracic, retroperitoneal or major orthopedic surgery (i.e., hip arthroplasty, internal fixation of hip or femur, hemipelvectomy, pelvic arthroplasty, knee arthroplasty, above-knee amputation or amputation below the knee but above the foot).
6. History of congestive heart failure
7. History of transient ischemic attack: a transient focal neurological deficit that lasted less than 24 hours and thought to be vascular in origin.
8. Hypertension: a physician diagnosis of hypertension.

**Exclusion criteria (any of)**

1. Active peptic ulcer disease
2. Thienopyridine (e.g., clopidogrel, ticlopidine, prasugrel) or ticagrelor within 72 hours prior to surgery; or intent to restart a thienopyridine or ticagrelor during the first 7 days post-operatively; or currently taking an alpha-2 agonist, alpha methyldopa, monoamino oxidase inhibitors or reserpine.
3. Planned use – during the first 3 days after surgery – therapeutic dose anticoagulation [e.g., dabigatran > 250 mg/day, rivaroxaban > 10 mg/day, or apixaban > 5 mg/day] or a therapeutic subcutaneous or intravenous antithrombotic agent (defined as full dose unfractionated heparin [i.e., > 15, 000 u/24 hours], low molecular weight heparin [i.e., > 6,000 u/24 hours or enoxaparin: > 60 mg/24 hours], or fondaparinux [i.e., > 2.5 mg/24 hours]).
4. Exception to Exclusion #3 for obese patients: Patients > 120 kg will be excluded if there is planned use (during the first 3 days after surgery): of Enoxaparin > 120 mg/24 hours or Dalteparin > 10,000 u/24 hours.
5. Did not undergo surgery (applies to this analysis only).
6. Underwent emergency/urgent surgery defined by surgeon booking the case for surgery to occur within 48 hours of acute presentation to hospital (applies to this analysis only).

# VISION variable definitions

## Outcome definitions

1. **MINS (myocardial injury after noncardiac surgery):** Defined as isolated ischemic myocardial injury or myocardial infarction.

*Isolated ischemic myocardial injury* included postoperative troponin elevation without evidence of non-ischemic etiology and without other signs or symptoms of ischemia.

A. Any peak non-high sensitivity cardiac troponin T ≥ 0.03 ng/mL resulting from myocardial ischemia (i.e., without evidence of a non-ischemic etiology) that occurred within the first 30 days after surgery based on non-high sensitivity cardiac troponin T using a Roche fourth-generation Elecsys assay, **OR**

B. Any peak postoperative high sensitivity cardiac troponin T measurement 20 to less than 65 ng/L with an absolute change of at least 5 ng/L, or at least 65 ng/L without evidence of a non-ischemic etiology that occurred with the first 30 days after surgery based on a Roche fifth-generation Elecsys assay.

**OR** myocardial infarction.

2. **Myocardial infarction:** Defined as any one of the following criteria (A, B or C) according to its universal definition:

A. A typical rise of troponin or a typical fall of an elevated troponin detected at its peak post surgery in a patient without a documented alternative explanation for an elevated troponin (e.g., pulmonary embolism). This criterion also required **that 1 of the following** must also exist:

i. ischemic signs or symptoms (i.e., chest, arm, neck or jaw discomfort; shortness of breath; pulmonary edema), **OR**

ii. development of pathologic Q waves present in any two contiguous leads that are ≥ 30 milliseconds, **OR**

iii. ECG changes indicative of ischemia (i.e., ST segment elevation [≥ 2 mm in leads V1, V2, or V3 OR ≥ 1 mm in the other leads], ST segment depression [≥ 1 mm], or symmetric inversion of T waves ≥ 1 mm) in at least two contiguous leads, **OR**

iv. coronary artery intervention (i.e., PCI or CABG surgery), **OR**

v. new or presumed new cardiac wall motion abnormality on echocardiography or new or presumed new fixed defect on radionuclide imaging.

B. Pathologic findings of an acute or healing myocardial infarction

C. Development of new pathological Q waves on an ECG if troponin levels were not obtained or were obtained at times that could have missed the clinical event.

3. **Nonfatal cardiac arrest:** Nonfatal cardiac arrest was defined as successful resuscitation from either documented or presumed ventricular fibrillation, sustained ventricular tachycardia, asystole, or pulseless electrical activity requiring cardiopulmonary resuscitation, pharmacological therapy, or cardiac defibrillation.

4. **Death due to a cardiac cause:** Death thought to be due to a cardiac cause including myocardial infarction‚ asystole, ventricular fibrillation, pulseless electrical activity, other sudden or arrhythmic death, sustained ventricular tachycardia, cardiogenic shock, congestive heart failure, or other cause thought to be cardiac in nature.

## Type of surgery performed

*If a patient underwent more than one surgery, all performed surgeries were included. If patients underwent any of the major surgical procedures, they were not classified as undergoing a ‘low risk surgery’.*

**Major Vascular Surgery**

1. Thoracic aorta reconstructive vascular surgeries (thoracic aortic aneurysm repair, repair of supra-aortic trunks not requiring total cardiopulmonary bypass, thoracoabdominal aortic aneurism repair with or without aorto-femoral bypass)

2. Aorto-iliac reconstructive vascular surgery (open abdominal aortic aneurysm repair, aorto-femoral bypass, iliac-femoral bypass, renal artery revascularization, celiac artery revascularization, superior mesenteric artery revascularization)

3. Peripheral vascular reconstruction without aortic cross-clamping (axillo-femoral bypass, femoral-femoral bypass, femoro-infragenicular bypass, profundoplasty, or other angioplasties of the infrainguinal arteries)

4. Extracranial cerebrovascular surgery (carotid endarterectomy, carotid-subclavian bypass)

5. EVAR – endovascular abdominal aortic aneurysm repair

**Major General Surgery**

1. Complex visceral resection (surgery involving the liver, esophagus, pancreas, or multiple organs)

2. Partial or total colectomy or stomach surgery

3. Other intra-abdominal surgery (gallbladder, appendix, adrenals, spleen, regional lymph node dissection)

4. Major head and neck resection for non-thyroid tumor

**Thoracic Surgery**

1. Pneumonectomy

2. Lobectomy

3. Other thoracic (wedge resection of lung, resection of mediastinal tumor, major chest wall resection)

**Major Urology or Gynecology Surgery**

1. Visceral resection (nephrectomy, ureterectomy, bladder resection, retroperitoneal tumor resection, exenteration [i.e. radical procedure for cancer to remove pelvic organs])

2. Cytoreductive surgery “debulking” done when cancer has spread in the pelvic/abdominal area, to remove as much of the tumor as possible

3. Radical hysterectomy is surgery to remove the uterus, cervix and part of the vagina

4. Hysterectomy is surgery to remove the uterus and usually the cervix

5. Radical prostatectomy is surgery to remove entire prostate gland and surrounding tissue

6. Transurethral prostatectomy to remove overgrowth of prostate tissue

**Major Orthopedic Surgery**

1. Major hip or pelvic surgery (hemi or total hip arthroplasty, internal fixation of hip, pelvic arthroplasty)

2. Internal fixation of femur

3. Knee arthroplasty

4. Above knee amputations

5. Lower leg amputation (amputation below knee but above foot)

**Major Neurosurgery**

1. Craniotomy

2. Major spine surgery is surgery involving multiple levels of the spine

**Other (Low Risk) Surgeries** (parathyroid, thyroid, breast, hernia, local anorectal procedure, oopherectomy, salpingectomy, endometrial ablation, peripheral nerve surgery, ophthalmology, ears/nose/throat surgery, vertebral disc surgery, hand surgery, cosmetic surgery, arterio-venous access surgery for dialysis, other surgeries).

**Urgent or emergency surgeries:** surgeries performed within 72 hours of acute event that led to need for surgery (excluded from this analysis).

## Patient factors

**Age:** the patient’s age in years, calculated as the difference between their birthdate and the date of surgery and rounded down to the nearest year.

**Requires assistance with activities of daily living:** patient requires assistance from another person with any of the following activities: dressing, eating, ambulating, toileting, hygiene**.** If a patient has suffered an acute injury leading to the need for surgery (e.g., hip fracture) the assessment for requirement of help for ADLs was based upon their condition prior to their acute injury.

**Congestive heart failure:** A physician diagnosis of a current or prior episode of congestive heart failure or prior radiographic evidence of vascular redistribution, interstitial pulmonary edema, or frank alveolar pulmonary edema.

**Coronary artery disease:** A current or prior history of any one of the following:

i. angina

ii. myocardial infarction or acute coronary syndrome

iii. a segmental cardiac wall motion abnormality on echocardiography or a segmental fixed defect on radionuclide imaging

iv. a positive radionuclide exercise, echocardiographic exercise, or pharmacological cardiovascular stress test demonstrating cardiac ischemia

v. coronary angiographic or CT coronary angiographic evidence of atherosclerotic stenosis ≥50% of the diameter of any coronary artery

vi. ECG with pathological Q waves in two contiguous leads

**History of stroke or transient ischemic attack:** A physician diagnosis of stroke, CT or MRI evidence of a prior stroke, or physician diagnosis of a prior transient ischemic attack.

**Peripheral arterial disease:** A current or prior history of: physician diagnosed intermittent claudication, vascular surgery for atherosclerotic disease, an ankle/arm systolic blood pressure ratio ≤ 0.90 in either leg at rest, or angiographic or doppler study demonstrating ≥ 70% stenosis in a noncardiac artery.

**Hypertension:** A physician diagnosis of hypertension.

**Chronic obstructive pulmonary disease (COPD):** If the chart or a physician has ever indicated that a patient has chronic bronchitis, we accepted this as a patient having COPD. If there is no mention of this but the patient reported they have had daily production of sputum for at least 3 months in 2 consecutive years then they were marked as having COPD. Likewise, if a physician has ever indicated that a patient has emphysema or if a patient's Pulmonary Function Tests state fixed or irreversible airflow limitation and/or emphysema then they were marked as having COPD.

**Diabetes not treated with insulin:** Patient states they have been diagnosed with diabetes or a physician has previously recorded that the patient has diabetes. This includes current gestational diabetes, but not past gestational diabetes that has resolved. The patient was not taking insulin prior to surgery.

**Diabetes treated with insulin:** Patient states they have been diagnosed with diabetes or a physician has previously recorded that the patient has diabetes. This includes current gestational diabetes, but not past gestational diabetes that has resolved. The patient was also taking insulin prior to surgery.

**Active cancer:** A patient was designated as having active cancer if they fulfilled any of the following criteria: i. undergoing surgery for cancer; ii. known metastatic disease; or iii. patient had received active treatment for their cancer (e.g., chemotherapy, radiation, or surgery) within the 6 months before their surgery, but this did not apply to patients with non-melanoma skin cancers or surgery for a biopsy.

**History of atrial fibrillation:** Patient has been diagnosed with atrial fibrillation by a physician.

**Preoperative hemoglobin**: The most recently measured preoperative serum concentration of hemoglobin.

**Preoperative estimated glomerular filtration rate:** calculated using the 2021 CKD-EPI creatinine equation based on the most recent preoperative serum creatinine.

# POISE-2 variable definitions

## Outcome definitions

1. **MINS (myocardial injury after noncardiac surgery):** Defined as isolated ischemic myocardial injury or myocardial infarction.

*Isolated ischemic myocardial injury* included postoperative cardiac marker elevation without evidence of nonischemic etiology and without other signs or symptoms of ischemia.

A. Any peak nonhigh sensitivity cardiac troponin T or troponin I ≥ 0.03 ng/mL resulting from myocardial ischemia (i.e. without evidence of a non-ischemic etiology) that occurred with the first 30 days after surgery based on non-high sensitivity cardiac troponin T or I assays, **OR**

B. Any peak postoperative high sensitivity cardiac troponin T measurement 20 to less than 65 ng/L with an absolute change of at least 5 ng/L, or at least 65 ng/L without evidence of a non-ischemic etiology that occurred with the first 30 days after surgery, **OR**

C. Any peak postoperative high sensitivity cardiac troponin I measurement at least 60 ng/L without evidence of a non-ischemic etiology that occurred within the first 30 days after surgery**, OR**

D. Any peak postoperative CK-MB greater than the upper limit of normal of the local laboratory without evidence of a non-ischemic etiology that occurred with the first 30 days after surgery**.**

Evidence of nonischemic etiology in POISE-2 included any of: sepsis 2 days before or 2 days after cardiac marker criteria were first met, pulmonary embolism 2 days before or 2 days after cardiac marker criteria were first met, or new clinically important atrial fibrillation 48 hours before or 48 hours after cardiac marker criteria were first met.

**OR** myocardial infarction.

2. **Myocardial infarction:** Defined as any one of the following criteria (A, B or C) according to its universal definition:

A. A typical rise of troponin or a typical fall of an elevated troponin detected at its peak post surgery in a patient without a documented alternative explanation for an elevated troponin (e.g., pulmonary embolism) OR a rapid rise and fall of CK-MB. This criterion also requires that 1 of the following must also exist:

i. ischemic signs or symptoms (i.e., chest, arm, neck or jaw discomfort; shortness of breath; pulmonary edema), **OR**

ii. development of pathologic Q waves present in any two contiguous leads that are ≥ 30 milliseconds, **OR**

iii. ECG changes indicative of ischemia (i.e., ST segment elevation [≥ 2 mm in leads V1, V2, or V3 OR ≥ 1 mm in the other leads], ST segment depression [≥ 1 mm], or symmetric inversion of T waves ≥ 1 mm) in at least two contiguous leads, **OR**

iv. coronary artery intervention (i.e., PCI or CABG surgery), **OR**

v. new or presumed new cardiac wall motion abnormality on echocardiography or new or presumed new fixed defect on radionuclide imaging.

B. Pathologic findings of an acute or healing myocardial infarction

C. Development of new pathological Q waves on an ECG if troponin levels were not obtained or were obtained at times that could have missed the clinical event.

3. **Nonfatal cardiac arrest:** Defined as successful resuscitation from either documented or presumed ventricular fibrillation, sustained ventricular tachycardia, asystole, or pulseless electrical activity requiring cardiopulmonary resuscitation, pharmacological therapy, or cardiac defibrillation.

4. **Death due to a cardiac cause:** Death thought to be due to a cardiac cause including myocardial infarction‚ asystole, ventricular fibrillation, pulseless electrical activity, other sudden or arrhythmic death, sustained ventricular tachycardia, cardiogenic shock, congestive heart failure, or other cause thought to be cardiac in nature.

## Type of surgery performed

**Major vascular surgery**: thoracic aortic aneurysm repair, repair of supra-aortic trunks not requiring total cardiopulmonary bypass, thoracoabdominal aortic aneurism repair with or without aorto-femoral bypass, open abdominal aortic aneurysm repair, aorto-femoral bypass, iliac-femoral bypass, renal artery revascularization, celiac artery revascularization, superior mesenteric artery revascularization, axillo-femoral bypass, femoral-femoral bypass, femoro-infragenicular bypass, profundoplasty, or other angioplasties of the infrainguinal arteries; extracranial cerebrovascular surgery (carotid endarterectomy, carotid-subclavian bypass), endovascular abdominal aortic aneurysm repair.

**Major thoracic surgery:** pneumonectomy, lobectomy, wedge resection of lung, resection of mediastinal tumor, major chest wall resection.

**Major orthopedic surgery:** hemi or total hip arthroplasty, internal fixation of hip, pelvic arthroplasty, hemipelvectomy, internal fixation of femur, knee arthroplasty, above-knee amputation, lower leg amputation below knee but above foot.

**Major urology/gynecology surgery:** nephrectomy, renal transplant, ureterectomy, bladder resection, retroperitoneal tumor resection, exenteration [i.e. radical procedure for cancer to remove pelvic organs]; cytoreductive surgery (“debulking” done when cancer has spread in the pelvic/abdominal area, to remove as much of the tumor as possible); radical hysterectomy to remove the uterus, cervix and part of the vagina; radical prostatectomy to remove entire prostate gland and surrounding tissue; hysterectomy to remove the uterus and usually the cervix; transurethral prostatectomy to remove overgrowth of prostate tissue.

**Major general surgery:** complex visceral resection involving the liver, esophagus, pancreas, or multiple organs; partial or total colectomy, or stomach surgery, small bowel resection; major head and neck resection for non-thyroid tumor; other intra-abdominal surgery such as gallbladder, appendix, adrenals, spleen, regional lymph node dissection.

**Major neurosurgery:** spine surgery involving multiple levels of the spine.

**Other (only low-risk) surgery**: parathyroid, thyroid, breast, hernia, local anorectal procedure, oopherectomy, salpingectomy, endometrial ablation, peripheral nerve surgery, ophthalmology, vertebral disc surgery, hand surgery, metatarsal resection, cosmetic surgery, arterio-venous access surgery for dialysis, shoulder or elbow arthroplasty, rotator cuff repair, internal fixation of humerus or tibia, knee or hip osteotomy, ankle fusion, or any other surgery not listed above.

**Open (versus endoscopic) surgical approach:** Endoscopic approaches include all endoscopic, laparoscopic, thorascopic, endovascular, and arthroscopic techniques.

**Urgent or emergency surgery:** surgeon books the case for surgery to occur within 48 hours of

acute presentation to hospital (excluded from these analyses)

## Patient factors

**Age:** the patient’s age in years, calculated as the difference between their birthdate and the date of surgery and rounded down to the nearest year.

**Congestive heart failure:** A physician diagnosis of a current or prior episode of congestive heart failure or prior radiographic evidence of vascular redistribution, interstitial pulmonary edema, or frank alveolar pulmonary edema.

**Coronary artery disease:** A current or prior history of any one of the following:

i. angina

ii. myocardial infarction or acute coronary syndrome

iii. a segmental cardiac wall motion abnormality on echocardiography or a segmental fixed defect on radionuclide imaging

iv. a positive radionuclide exercise, echocardiographic exercise, or pharmacological cardiovascular stress test demonstrating cardiac ischemia

v. coronary angiographic or CT coronary angiographic evidence of atherosclerotic stenosis ≥50% of the diameter of any coronary artery

vi. ECG with pathological Q waves in two contiguous leads

**History of stroke or transient ischemic attack:** A physician diagnosis of stroke, CT or MRI evidence of a prior stroke, or physician diagnosis of a prior transient ischemic attack.

**Peripheral arterial disease:** A current or prior history of: physician diagnosed intermittent claudication, vascular surgery for atherosclerotic disease, an ankle/arm systolic blood pressure ratio ≤ 0.90 in either leg at rest, or angiographic or doppler study demonstrating ≥ 70% stenosis in a noncardiac artery.

**Hypertension:** A physician diagnosis of hypertension.

**Diabetes treated with oral agent or with insulin:** Patient states they have been diagnosed with diabetes or a physician has previously recorded that the patient has diabetes. This includes current gestational diabetes, but not past gestational diabetes that has resolved. The patient is treated with an oral agent or with insulin.

**Preoperative atrial fibrillation:** ECG evidence of atrial fibrillation before surgery.

**Preoperative dialysis:** Defined as the use of a hemodialysis machine or peritoneal dialysis apparatus in the 7 days before surgery.

**Preoperative hemoglobin**: The most recently measured preoperative serum concentration of hemoglobin.

**Preoperative estimated glomerular filtration rate:** calculated using the 2021 CKD-EPI creatinine equation based on the most recent preoperative serum creatinine.

# Table S1. VISION participant characteristics.

|  | N = 35,815 |
| --- | --- |
| Age, years | 63 (54 – 72) |
| Female | 17,767 (49.6) |
| Male | 18,048 (50.4) |
| Preop. hemoglobin, g/L | 132 (120 –144) |
| Preop. estimated glomerular filtration rate, mL/min per 1.73 m^2^ | 89 (71 – 101) |
| Preop. dialysis | 485 (1.4) |
| History of hypertension | 18,217 (50.9) |
| History of coronary artery disease | 4,730 (13.2) |
| History of peripheral arterial disease | 2,910 (8.1) |
| History of stroke or transient ischemic attack | 2,225 (6.2) |
| History of congestive heart failure | 1,243 (3.5) |
| History of atrial fibrillation | 1,148 (3.2) |
| Diabetes |  |
| Diabetes with no preoperative insulin | 4,874 (13.6) |
| Diabetes with preoperative insulin | 2,744 (7.7) |
| Active cancer | 9,423 (26.3) |
| Requires help with activities of daily living | 1,793 (5.0) |
| History of chronic obstructive pulmonary disease | 2,860 (8.0) |
| Thoracic aorta reconstruction | 71 (0.2) |
| Aorto-iliac reconstruction | 599 (1.7) |
| Peripheral vascular reconstruction without aortic cross-clamping | 1,145 (3.2) |
| Extracranial cerebrovascular surgery | 423 (1.2) |
| Endovascular aneurysm repair | 292 (0.8) |
| Complex visceral resection | 1,107 (3.1) |
| Partial or total colectomy or stomach surgery | 2,002 (5.6) |
| Other abdominal surgery | 3,339 (9.3) |
| Major head and neck resection of non-thyroid tumor | 633 (1.8) |
| Pneumonectomy | 43 (0.1) |
| Lobectomy | 457 (1.3) |
| Other thoracic surgery | 640 (1.8) |
| Visceral resection | 1,046 (2.9) |
| Urologic or gynecologic visceral resection or cytoreduction | 274 (0.8) |
| Subtotal hysterectomy | 1,347 (3.8) |
| Radical hysterectomy | 437 (1.2) |
| Radical prostatectomy | 720 (2.0) |
| Transurethral prostatectomy | 990 (2.8) |
| Major hip or pelvic surgery | 2,373 (6.6) |
| Internal fixation of femur | 435 (1.2) |
| Knee arthroplasty | 2,841 (7.9) |
| Above-knee amputation | 172 (0.5) |
| Below-knee amputation | 202 (0.6) |
| Craniotomy | 749 (2.1) |
| Major spine surgery | 1358 (3.8) |
| Other (only low-risk) surgery | 12,857 (35.9) |
| Open surgery | 27,723 (77.5) |

Footnote: Continuous variables are summarized as median (25^th^ percentile – 75^th^ percentile); categorical variables are summarized as count (% of study total).

# Table S2. POISE-2 participant characteristics.

|  | ****N = 9,219**** |
| --- | --- |
| Age, years | 70 (61 – 76) |
| Female | 4,310 (46.8) |
| Male | 4,909 (53.2) |
| Preop. hemoglobin, g/L | 133 (121 – 144) |
| Preop. estimated glomerular filtration rate, mL/min per 1.73 m^2^ | 82 (64 – 94) |
| Dialysis in the week before surgery | 108 (1.2) |
| History of hypertension | 8,098 (87.8) |
| History of coronary artery disease | 2,167 (23.5) |
| History of peripheral arterial disease | 807 (8.8) |
| History of stroke or transient ischemic attack | 802 (8.7) |
| History of congestive heart failure | 305 (3.3) |
| Preop. atrial fibrillation | 213 (2.3) |
| History of diabetes treated with oral medications or insulin | 3,562 (38.6) |
| Major orthopedic surgery | 3,283 (35.6) |
| Other orthopedic surgery | 251 (2.7) |
| Major general surgery | 1,692 (18.4) |
| Other general surgery | 721 (7.8) |
| Major urology/gynecology surgery | 1,190 (12.9) |
| Other urology/gynecology surgery | 473 (5.1) |
| Major thoracic surgery | 394 (4.3) |
| Other thoracic surgery | 198 (2.1) |
| Major vascular surgery | 477 (5.2) |
| Other vascular surgery | 118 (1.3) |
| Major spine surgery | 193 (2.1) |
| Other (only low-risk) surgery | 413 (4.5) |
| Open surgery | 7,126 (77.3) |

Footnote: Continuous variables are summarized as median (25^th^ percentile – 75^th^ percentile); categorical variables are summarized as count (% of study total).

# Figure S1. Internal validation of the primary VISION prediction model.

**
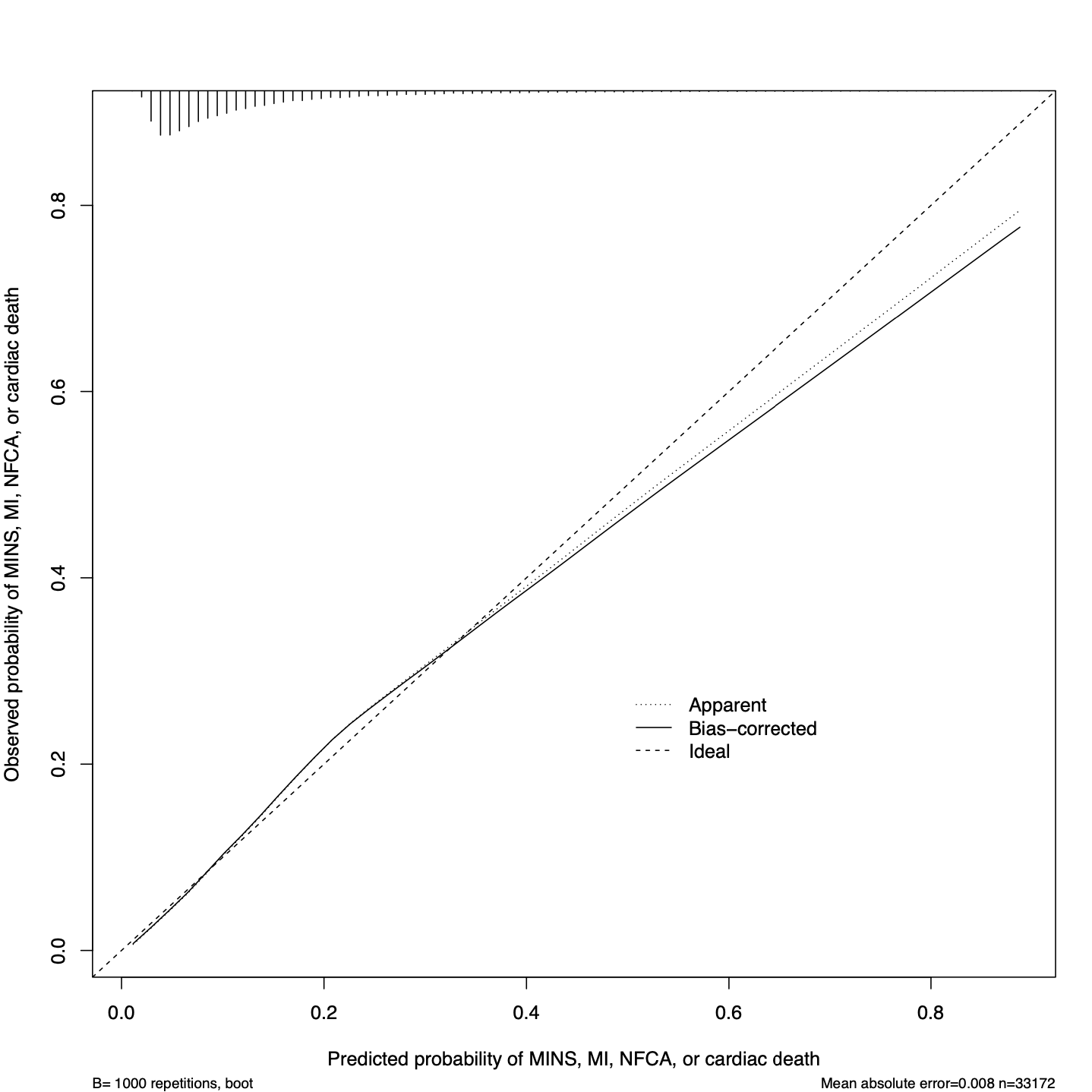
**

**Footnote:** Performance evaluation of the full logistic model in complete-case data in the VISION cohort showed excellent bias-corrected calibration without evidence of meaningful overfitting. The bias-corrected calibration slope was 0.98 (95% CI, 0.98-0.98), and bias-corrected intercept = -0.03 (95% CI, -0.03 to -0.02). Discrimination was also acceptable with a bias-corrected C-statistic of 0.77 (95% CI, 0.76-0.77). Bias corrections were obtained through bootstrap validation in 1000 samples; confidence intervals were derived by repeating the internal validation bootstrap procedure in 200 samples and calculating the 2.5^th^ and 97.5^th^ percentiles of each metric; the point estimate was the median. **Abbreviations:** MINS, myocardial injury after noncardiac surgery; MI, myocardial infarction; NFCA, nonfatal cardiac arrest; VISION, Vascular events In noncardiac Surgery patIents cOhort evaluatioN.

# Figure S2. Internal validation of the primary POISE-2 prediction model.

**
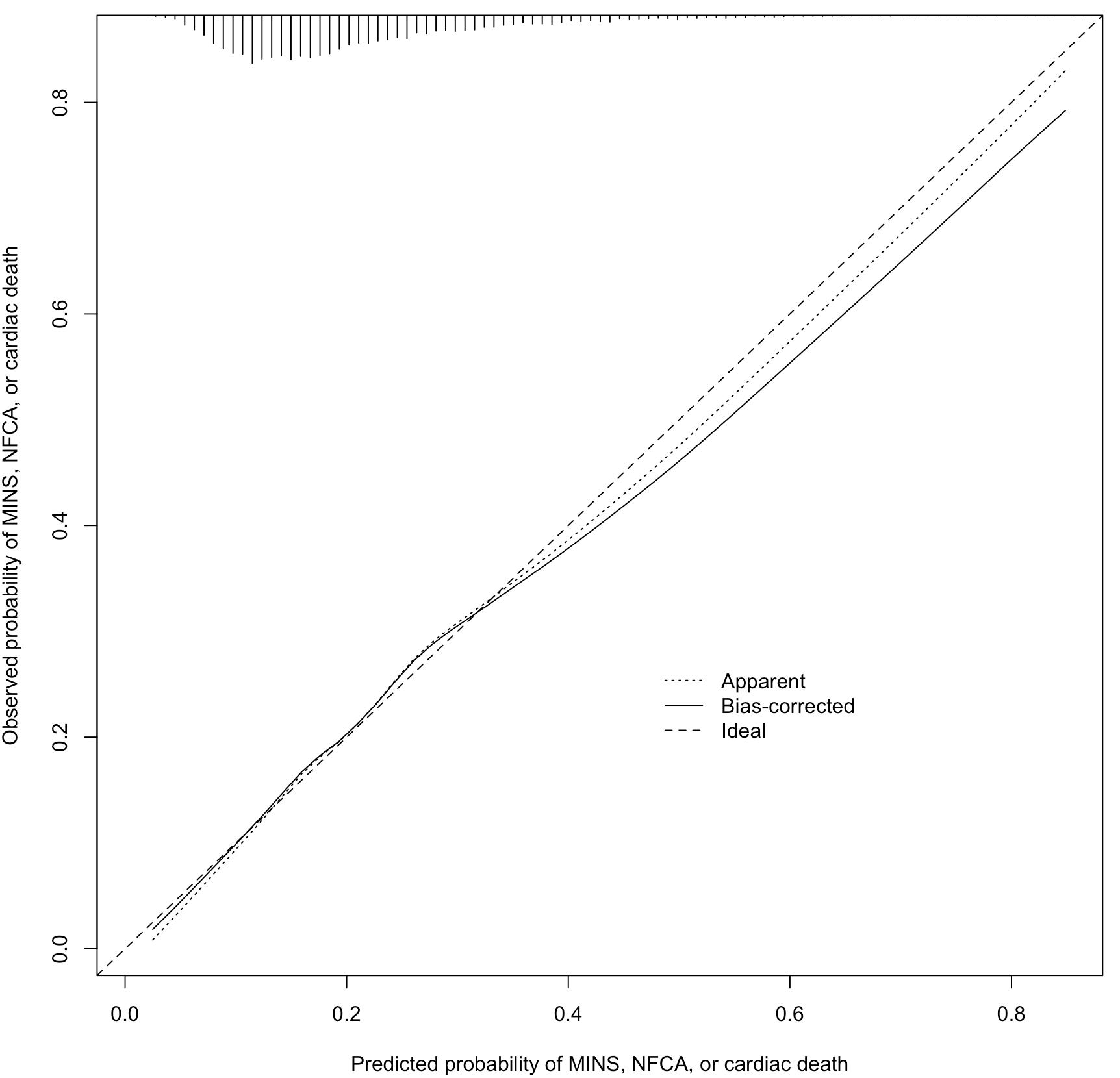
**

**Footnote:** Performance evaluation of the full logistic model fit in in 8,890 patients with complete data who underwent nonurgent, nonemergency, noncardiac surgery in the POISE-2 trial showed excellent bias-corrected calibration without evidence of meaningful overfitting. The bias-corrected calibration slope was 0.95 (95% CI, 0.94-0.95), and bias-corrected intercept = -0.06 (95% CI, -0.08 to -0.05). Discrimination was limited, with a bias-corrected C-statistic of 0.69 (95% CI, 0.68-0.70). Bias corrections were obtained through bootstrap validation in 1000 samples; confidence intervals were derived by repeating the internal validation bootstrap procedure in 200 samples and calculating the 2.5^th^ and 97.5^th^ percentiles of each metric; the point estimate was the median value. **Abbreviations:** MINS, myocardial injury after noncardiac surgery; MI, myocardial infarction; NFCA, nonfatal cardiac arrest; POISE-2, PeriOperative Ischemic Evaluation-2.

# Figure S3. Sensitivity analyses of age-and-sex-specific relationship between preoperative eGFR and perioperative cardiac events.

**
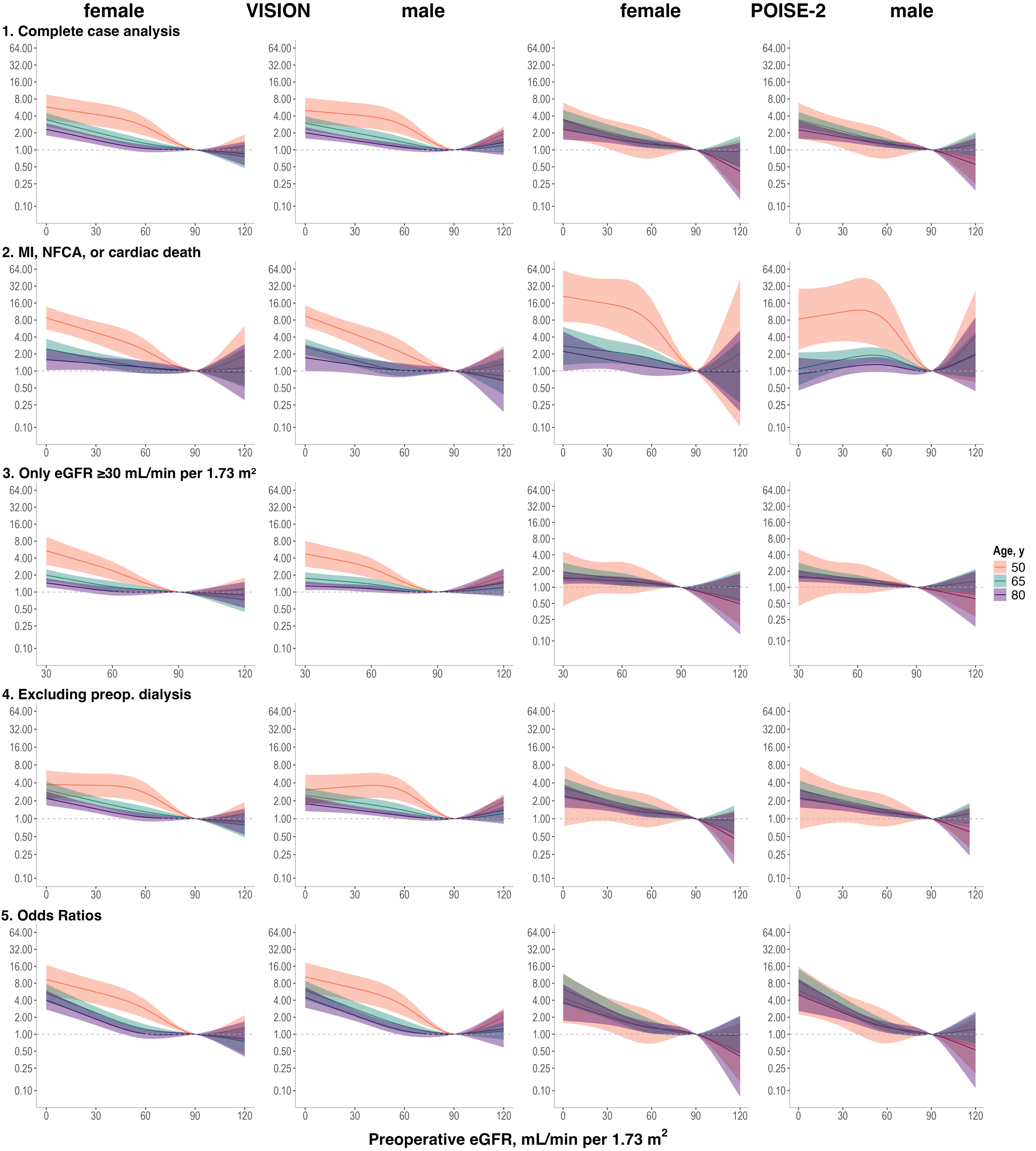
**

**Footnote:** The reference eGFR concentration is 90 mL/min per 1.73 m^2^. The shaded regions represent 95% confidence intervals adjusted for clustering by study center. The Y-axis refers to adjusted relative risk in the first three sensitivity analyses and odds ratio in the fourth. In **sensitivity analysis 1**, the primary analysis of the composite 30-day outcome including MINS, nonfatal cardiac arrest, or death due to cardiac cause was repeated only in patients with complete predictor data: VISION, n = 33,171; 4,473 events; p < 0.001 for relationship between eGFR and 30-day cardiac events that was not linear (p_nonlinearity_ <0.001) and differed by age (p_interaction_ <0.001) and sex (p_interaction_ = 0.02). In POISE-2, n = 8,893, 1,850 events; p < 0.001 for relationship; p_nonlinearity_ = 0.36; p_age-eGFR-interaction_ = 0.02; p_sex-eGFR-interaction_ = 0.78. These results were consistent with the primary analysis that relied on multiple imputation of missing data. In **sensitivity analysis 2**, with outcome changed to a composite of myocardial infarction, nonfatal cardiac arrest, or death due to a cardiac cause: VISION, n = 33,172; 1,111 events; p <0.001 for relationship; p_nonlinearity_ <0.001; p_age-eGFR-interaction_ <0.001; p_sex-eGFR-interaction_ = 0.76; POISE-2, n = 8,893; 601 events; p<0.001 for relationship; p_nonlinearity_ =0.002; p_age-eGFR-interaction_ <0.001; p_sex-eGFR-interaction_ = 0.09. In **sensitivity analysis 3**, we included only patients with eGFR≥ 30 mL/min per 1.73 m^2^: VISION, n = 32,073; 3,941 events; p <0.001 for relationship, p_nonlinearity_ <0.001, p_age-eGFR-interaction_ <0.001, p_sex-eGFR-interaction_ = 0.09; POISE-2, n = 8,599; 1,703 events; p <0.001 for relationship, p_nonlinearity_ = 0.41, p_age-eGFR-interaction_ = 0.28, p_sex-eGFR-interaction_ = 0.77. **Sensitivity analysis 4** included only patients with complete data who were not receiving dialysis before surgery: VISION, n = 32,697; 4,188 events; p <0.001 for relationship, p_nonlinearity_ <0.001, p_age-eGFR-interaction_ <0.001, p_sex-eGFR-interaction_ = 0.053; POISE-2, n = 8,788; 1,790 events; p <0.001 for relationship, p_nonlinearity_ = 0.50, p_age-eGFR-interaction_ = 0.049, p_sex-eGFR-interaction_ = 0.79. In **sensitivity analysis 5,** we used logistic regression models to obtain odds ratios instead of relative risks in 35,815 patients in VISION and 9,219 patients in POISE-2 after multiple imputation of missing data. In VISION, eGFR was associated with the composite of MINS, nonfatal cardiac arrest, or death due to a cardiac cause (p <0.001; p_nonlinearity_ = 0.001) and the relationship differed by age (p_interaction_ <0.001) but not sex (p_interaction_ = 0.32). In POISE-2, eGFR was similarly associated with the primary composite (p <0.001; p_nonlinearity_ = 0.01) and the relationship did not significantly differ by age (p_interaction_ = 0.17) or sex (p_interaction_ =0.44). **Abbreviations:** MINS, myocardial injury after noncardiac surgery; MI, myocardial infarction; NFCA, nonfatal cardiac arrest; eGFR, estimated glomerular filtration rate; VISION, Vascular events In noncardiac Surgery patIents cOhort evaluatioN; POISE-2, PeriOperative Ischemic Evaluation-2.

# Figure S4. Predictor importance for composite outcome of myocardial infarction, nonfatal cardiac arrest, or death due to cardiac cause.

**
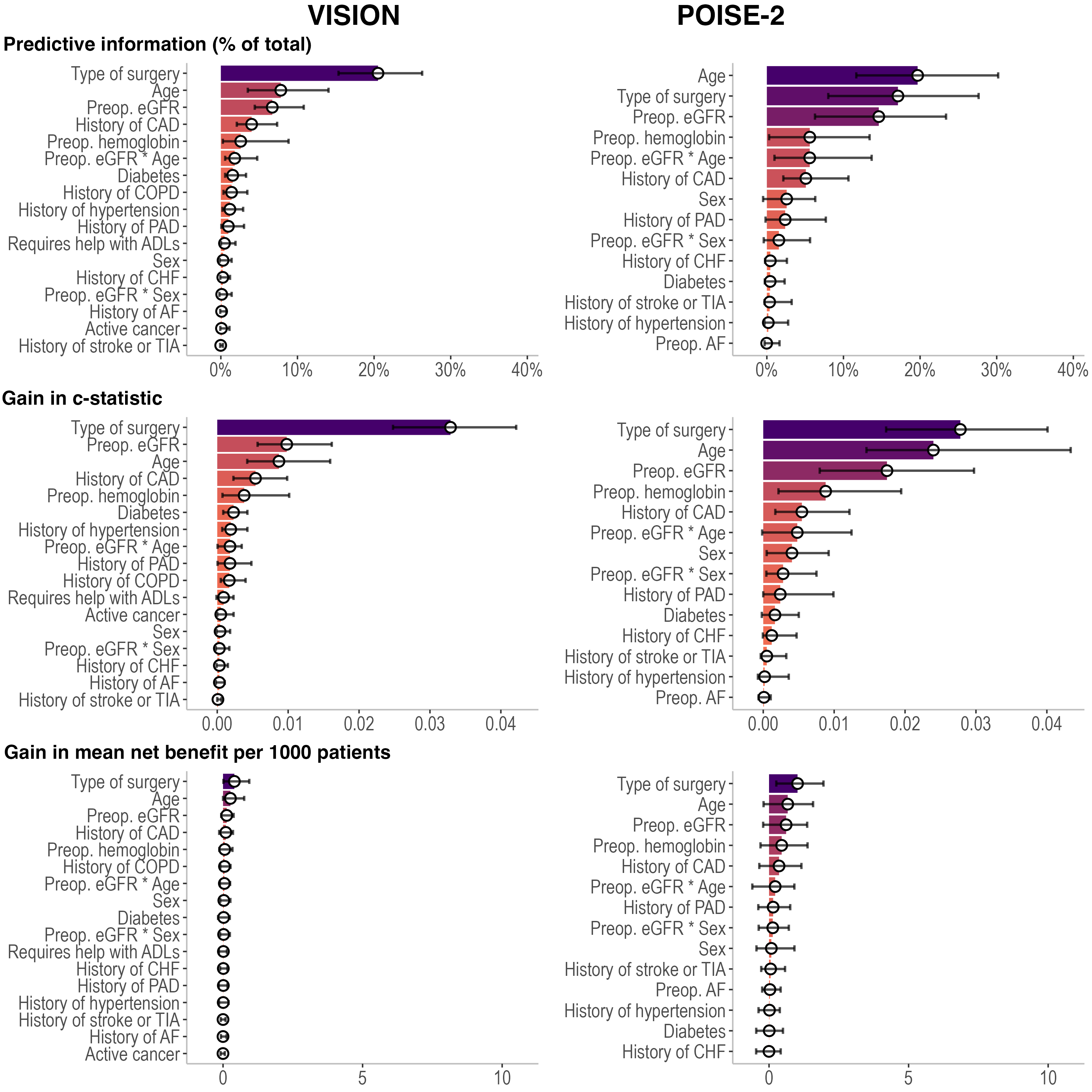
**

**Footnote:** Percentage of total predictive information, gain in c-statistic, and gain in mean net benefit contributed by individual predictors from logistic regression models predicting a composite of myocardial infarction, nonfatal cardiac arrest, or death due to cardiac cause occurring within 30 days after elective noncardiac surgery in patients without missing data in VISION (n=33,171) and POISE-2 (n=8,893). The contributions from all surgical variables are summed in a single ‘Type of Surgery’ item for clarity. Point estimates (medians) and 95% confidence intervals are derived from 1,000 cluster-based bootstrap samples. Abbreviations: MINS, myocardial injury after noncardiac surgery; eGFR, estimated glomerular filtration rate; ADLs, activities of daily living; CAD, coronary artery disease; PAD, peripheral arterial disease; COPD, chronic obstructive pulmonary disease; AF, atrial fibrillation; CHF, congestive heart failure, TIA, transient ischemic attack; VISION, Vascular events In noncardiac Surgery patIents cOhort evaluatioN; POISE-2, PeriOperative Ischemic Evaluation-2.

# Figure S5. Predictor importance restricted to patients with eGFR ≥ 30 ml/min per 1.73 m^2^.

**
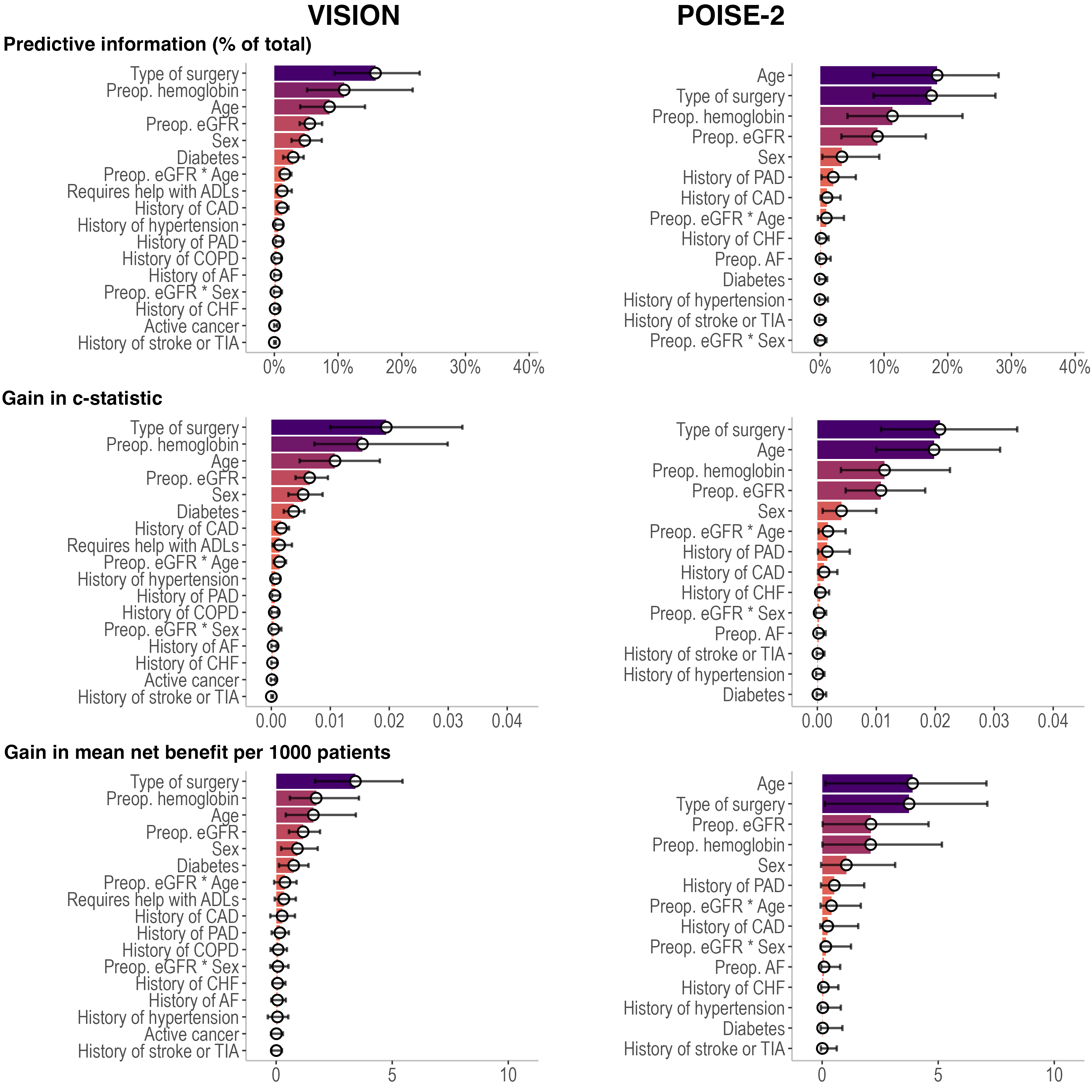
**

**Footnote:** Percentage of total predictive information, gain in c-statistic, and gain in mean net benefit contributed by individual predictors from logistic regression models predicting a composite of myocardial infarction, nonfatal cardiac arrest, or death due to cardiac cause occurring within 30 days after elective noncardiac surgery in patients without missing data and who had eGFR ≥ 30 mL/min per 1.73 m^2^ in VISION (n=32,073) and POISE-2 (n=8,599). The contributions from all surgical variables are summed in a single ‘Type of Surgery’ item for clarity. Point estimates (medians) and 95% confidence intervals are derived from 1,000 cluster-based bootstrap samples. Abbreviations: MINS, myocardial injury after noncardiac surgery; eGFR, estimated glomerular filtration rate; ADLs, activities of daily living; CAD, coronary artery disease; PAD, peripheral arterial disease; COPD, chronic obstructive pulmonary disease; AF, atrial fibrillation; CHF, congestive heart failure, TIA, transient ischemic attack; VISION, Vascular events In noncardiac Surgery patIents cOhort evaluatioN; POISE-2, PeriOperative Ischemic Evaluation-2.

# Figure S6. Predictor importance restricted to patients not receiving dialysis before surgery.


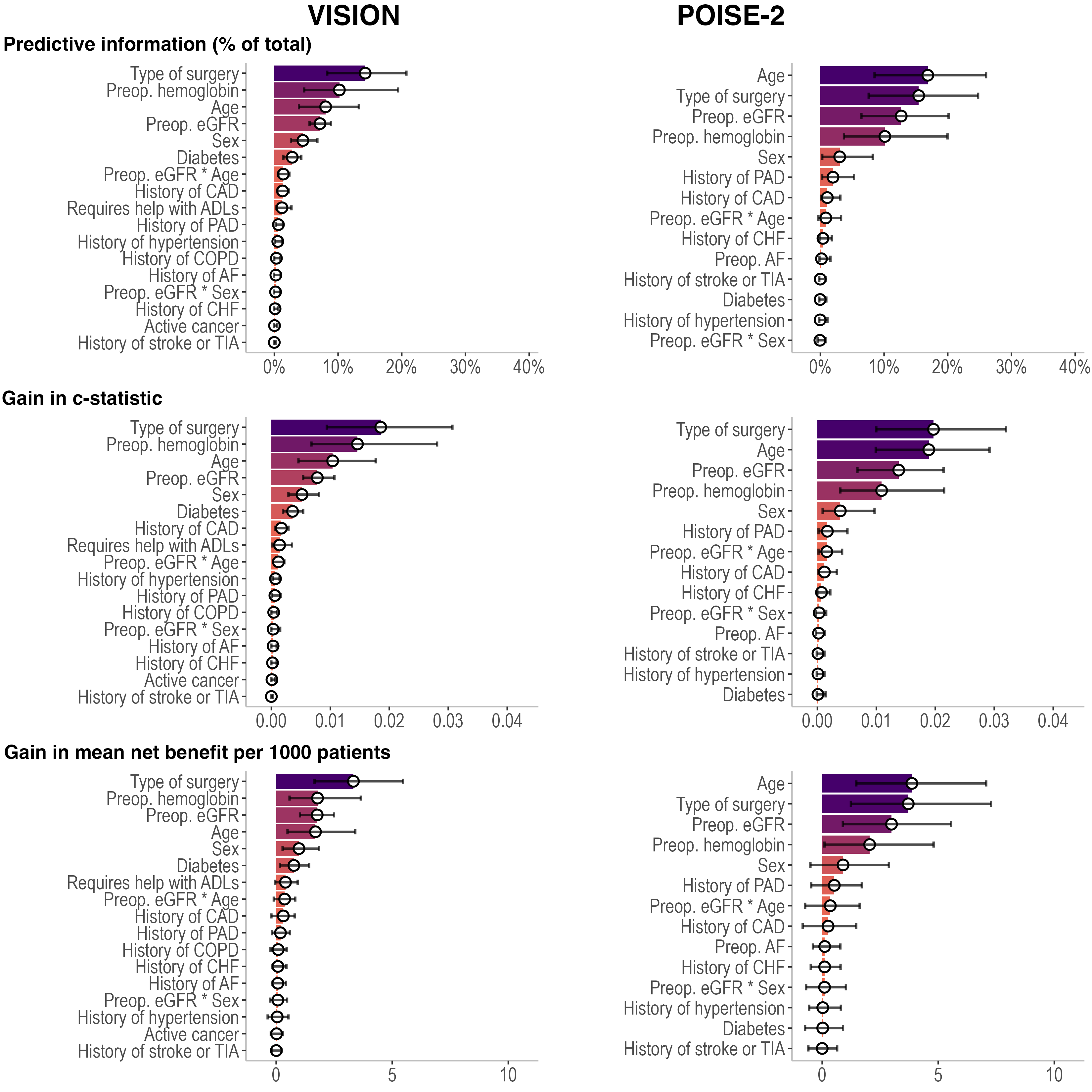


**Footnote:** Percentage of total predictive information, gain in c-statistic, and gain in mean net benefit contributed by individual predictors from logistic regression models predicting a composite of myocardial infarction, nonfatal cardiac arrest, or death due to cardiac cause occurring within 30 days after elective noncardiac surgery in patients without missing data and who were not receiving dialysis before surgery in VISION (n=32,697) and POISE-2 (n=8,788). The contributions from all surgical variables are summed in a single ‘Type of Surgery’ item for clarity. Point estimates (medians) and 95% confidence intervals are derived from 1,000 cluster-based bootstrap samples. Abbreviations: MINS, myocardial injury after noncardiac surgery; eGFR, estimated glomerular filtration rate; ADLs, activities of daily living; CAD, coronary artery disease; PAD, peripheral arterial disease; COPD, chronic obstructive pulmonary disease; AF, atrial fibrillation; CHF, congestive heart failure, TIA, transient ischemic attack; VISION, Vascular events In noncardiac Surgery patIents cOhort evaluatioN; POISE-2, PeriOperative Ischemic Evaluation-2.

# Figure S7. Relationship between predicted probability of cardiac events with versus without eGFR in the model, by sex.

**
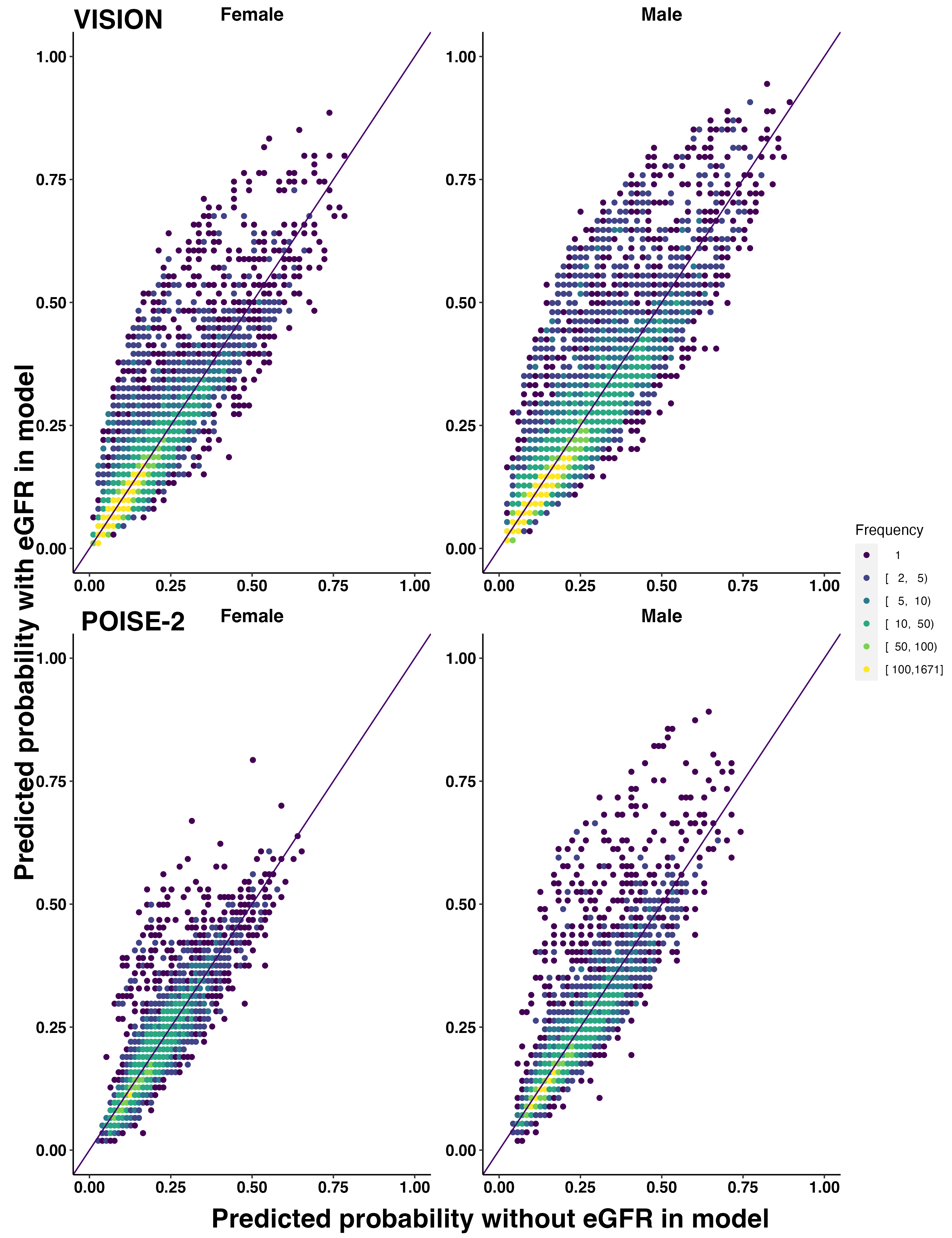
**

**Footnote:** Outcome is composite of MINS, nonfatal cardiac arrest, or death due to cardiac cause. This figure demonstrates the extent to which adding eGFR to the models to predict MINS, nonfatal cardiac arrest, or cardiac death affects predicted probabilities. Abbreviations: eGFR, estimated glomerular filtration rate; MINS, myocardial injury after noncardiac surgery; VISION, Vascular events In noncardiac Surgery patIents cOhort evaluatioN; POISE-2, PeriOperative Ischemic Evaluation-2.

# Figure S8. Change in predicted probability of perioperative cardiac events with addition of eGFR to the model across the range of eGFR, by sex.

**
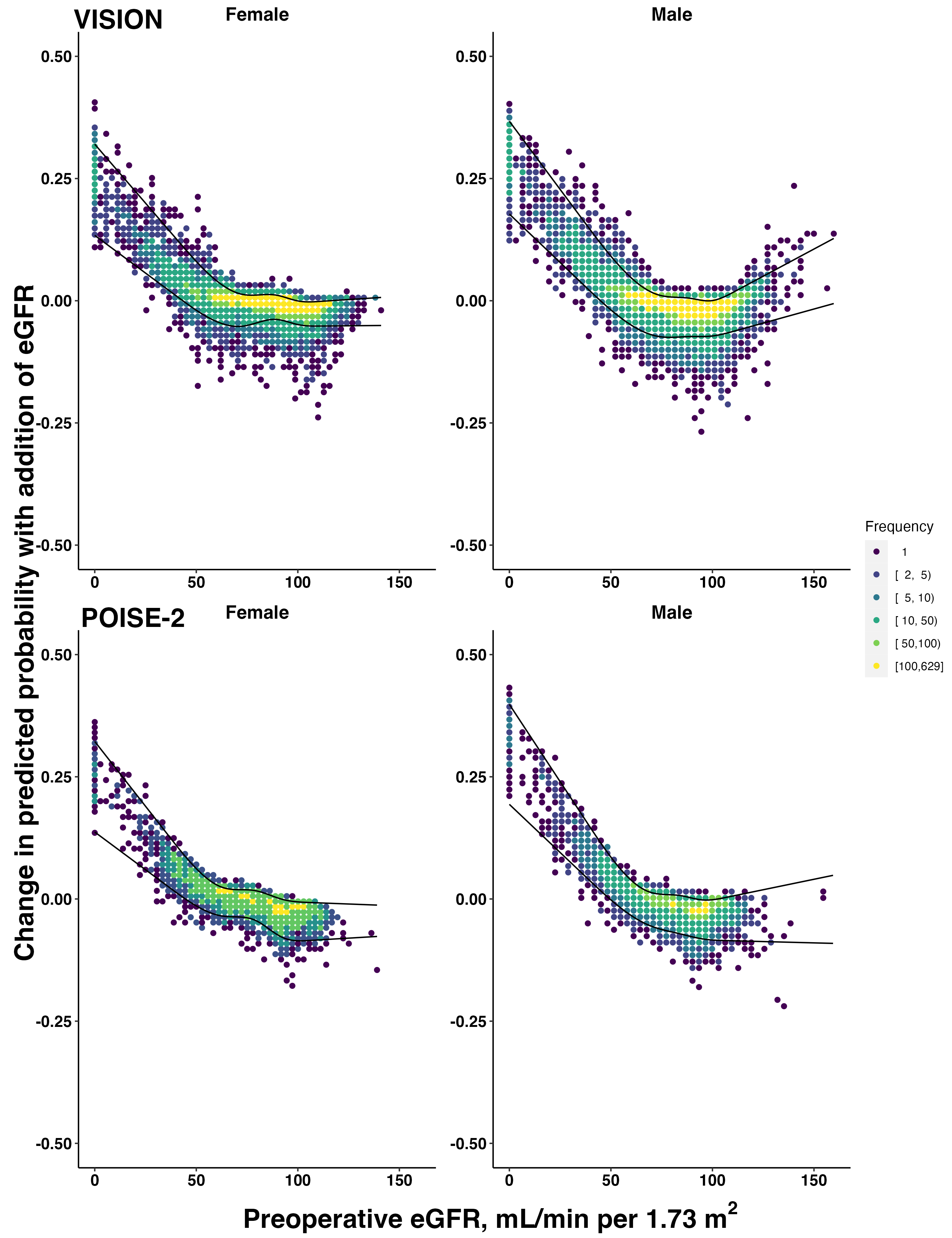
**

**Footnote:** Outcome is composite of MINS, nonfatal cardiac arrest, or death due to cardiac cause. The bands indicate the region representing 90% of participants. Accounting for eGFR when eGFR <50 mL/min per 1.73m^2^ substantially increases the predicted risk values while moderately reducing the predicted risk values for a small proportion of participants with eGFR >50 mL/min per 1.73m^2^. For a small proportion of male participants in VISION, eGFR >120 increased risk estimates moderately which may explain the statistically significant interaction between eGFR and sex found in VISION but not found in POISE-2. Abbreviations: eGFR, estimated glomerular filtration rate; MINS, myocardial injury after noncardiac surgery; VISION, Vascular events In noncardiac Surgery patIents cOhort evaluatioN; POISE-2, PeriOperative Ischemic Evaluation-2.

# Figure S9. Change in predicted probability of perioperative cardiac events with addition of eGFR to the model across age, by sex.

**
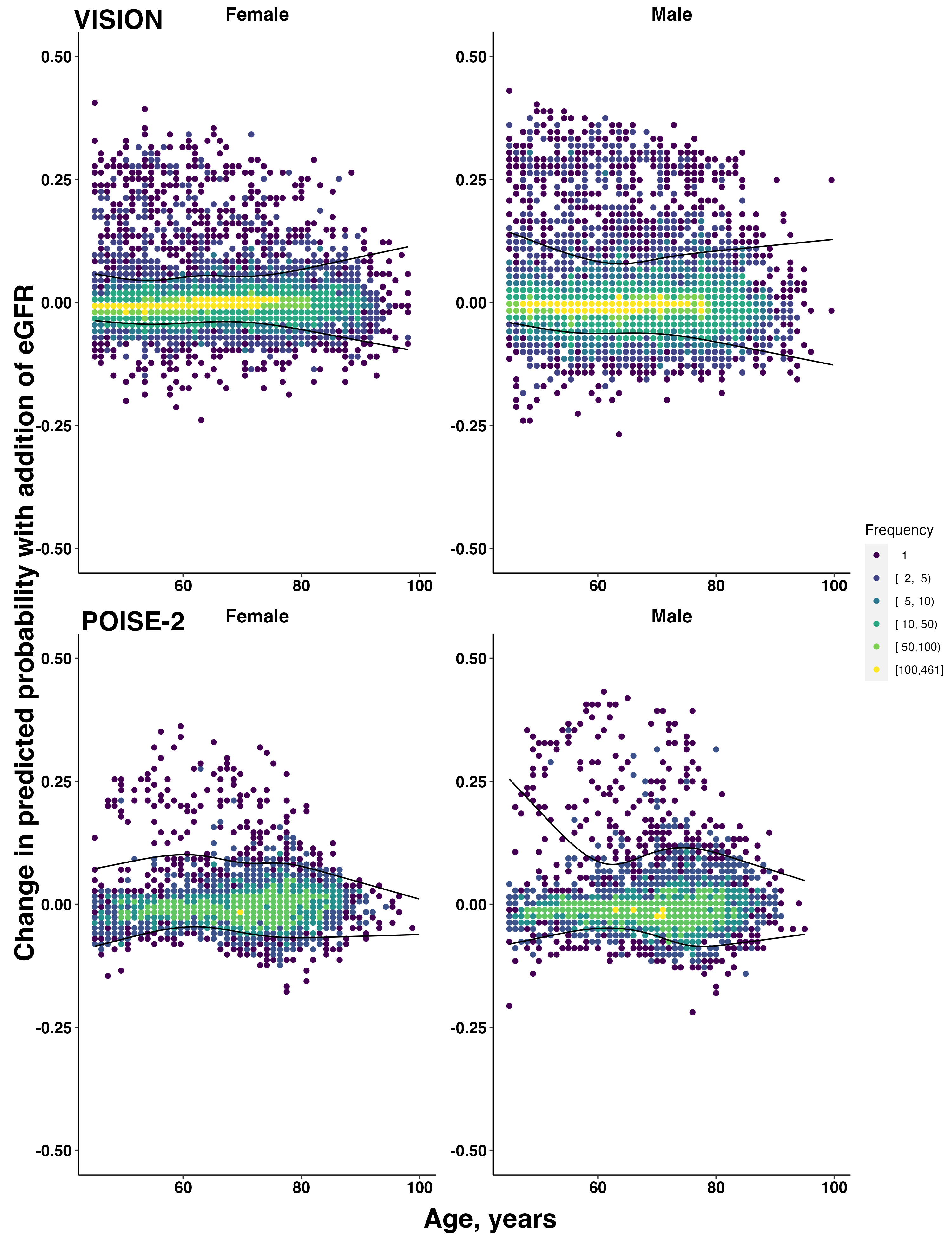
**

**Footnote:** Outcome is composite of MINS, nonfatal cardiac arrest, or death due to cardiac cause. The bands contain the region representing 90% of participants. Although there was a statistically significant interaction between eGFR and age in both studies, the distribution of change in predicted probability when eGFR is added to the model is largely consistent across age in both studies until approximately age 80. After age 80, accounting for eGFR in the model rarely makes a large change to predicted probability. Abbreviations: eGFR, estimated glomerular filtration rate; MINS, myocardial injury after noncardiac surgery; VISION, Vascular events In noncardiac Surgery patIents cOhort evaluatioN; POISE-2, PeriOperative Ischemic Evaluation-2.
